# Palm Fruit Bioactives modulate human astrocyte activity *in vitro* altering the cytokine secretome reducing levels of TNFα, RANTES and IP-10

**DOI:** 10.1038/s41598-018-34763-3

**Published:** 2018-11-06

**Authors:** Robert P. Weinberg, Vera V. Koledova, Kirsten Schneider, T. G. Sambandan, Adlai Grayson, Gal Zeidman, Anastasia Artamonova, Ravigadevi Sambanthamurthi, Syed Fairus, Anthony J. Sinskey, ChoKyun Rha

**Affiliations:** 1 Department of Biology, Massachusetts Institute of Technology, Cambridge, MA 02139 USA; 2 Biomaterials Science and Engineering Laboratory, Massachusetts Institute of Technology, Cambridge, MA 02139 USA; 3 Advanced Biotechnology and Breeding Centre, Malaysian Palm Oil Board, 6, Persiaran Institusi, Bandar Baru Bangi, 43000 Kajang, Selangor Malaysia

## Abstract

Neurodegenerative diseases, including Alzheimer’s disease and Parkinson’s disease, are becoming more prevalent and an increasing burden on society. Neurodegenerative diseases often arise in the milieu of neuro-inflammation of the brain. Reactive astrocytes are key regulators in the development of neuro-inflammation. This study describes the effects of Palm Fruit Bioactives (PFB) on the behavior of human astrocytes which have been activated by IL-1β. When activated, the astrocytes proliferate, release numerous cytokines/chemokines including TNFα, RANTES (CCL5), IP-10 (CXCL10), generate reactive oxygen species (ROS), and express specific cell surface biomarkers such as the Intercellular Adhesion Molecule (ICAM), Vascular Cellular Adhesion Molecule (VCAM) and the Neuronal Cellular Adhesion Molecule (NCAM). Interleukin 1-beta (IL-1β) causes activation of human astrocytes with marked upregulation of pro-inflammatory genes. We show significant inhibition of these pro-inflammatory processes when IL-1β-activated astrocytes are exposed to PFB. PFB causes a dose-dependent and time-dependent reduction in specific cytokines: TNFα, RANTES, and IP-10. We also show that PFB significantly reduces ROS production by IL-1β-activated astrocytes. Furthermore, PFB also reduces the expression of ICAM and VCAM, both in activated and naïve human astrocytes *in vitro*. Since reactive astrocytes play an essential role in the neuroinflammatory state preceding neurodegenerative diseases, this study suggests that PFB may have a potential role in their prevention and/or treatment.

## Introduction

The normal aging process is associated with some inflammation in the brain and this neuro-inflammation often precedes neurodegenerative conditions^1–3^. Neuroinflammation is a prominent characteristic of multiple neurodegenerative disorders including multiple sclerosis (MS), Alzheimer’s disease (AD), Parkinson’s disease (PD), Huntington’s disease (HD) and Autism Spectrum Disorder (ASD)^4–8^. Epidemiologic studies reveal that the global burden of these neurodegenerative diseases is increasing^9–11^.

Astrocytes are innate, immune, star-shaped glial cells found in the CNS, which upon activation, proliferate and produce cytokines and other inflammatory mediators^12^. Astrocytes are the most abundant glial cell type in the brain. They provide important functions including nutritional support of metabolites and growth factors to neurons, supporting the formation and plasticity of synapses, controlling immune cell activation and trafficking, and regulating the extracellular fluid environment of ions and neurotransmitters^13,14^. Following traumatic injury, the astrocytes are involved in wound healing processes^15,16^.

Current theories of neurodegenerative diseases have shifted the traditional focus from neurons to the role of the astrocytes^17–19^. The signaling pathways involving the cytokines and chemokines become dysregulated in neurodegenerative diseases^20^. These dysregulated signaling pathways have a major pathologic effect on the intricate network of astrocyte-neuron interactions and system dynamics^12,14,17–21^. CNS tissue damage often results from the dysregulated microglia and astrocytes^21^. Astrocytes comprise part of the innate immune system, able to detect danger signals with toll-like cell surface receptors and respond with the secretion of cytokines and chemokines which then may recruit lymphocytes and result in the subsequent activation of the adaptive immune system^22,23^.

Multiple types of injury to the brain will cause a neuro-inflammatory response including infection, trauma and toxins^24^. The endogenous cells mediating neuroinflammation in the brain are the microglia and astrocytes. Depending on the type of insult, microglia and astrocytes have both pro-inflammatory and anti-inflammatory actions^25–31^. Resident microglia and infiltrating immune cells together drive astrocyte activation and reactivity^31^. Astrocytes play a key role in the pathogenesis of neurodegenerative diseases^31–36^. The astrocyte response repertoire includes activation of the adaptive immune system, neutralizing microbial pathogens, phagocytosis of dead cells and debris, secretion of neurotrophins, repair of the Blood Brain Barrier, phagocytosis of synapses, and encircling scar formation to restrict the expansion of infection or hematoma^26,30,31,37–42^. The blood-brain barrier (BBB) restricts the usual circulating leukocyte response to inflammation in the brain creating a unique “immune-privileged” territory in the brain^13,22,23,29^. This limitation restricts the access of many immune cells along with their associated cytokines and reactive oxygen species.

Reactive astrocytes were morphologically first described in the 1970s with the discovery of the intermediate filament protein GFAP (Glial Fibrillary Acidic Protein)^43^. Anti-GFAP antibodies then allowed immunostaining of human glial tumors and GFAP quantitation using rocket electrophoresis^44,45^. Then with the molecular cloning of mouse *Gfap*, GFAP became the standard biomarker of reactive astrocytes^46^. However further experimental studies revealed marked heterogeneity of GFAP in astrocytes under varying conditions and even within the same brain^25,31,33,44,47–50^.

Analogous to the pro-inflammatory M1 and anti-inflammatory M2 phenotypic states for macrophages, transcriptome analysis reveals that there are two classes of reactive astrocytes called A1 and A2^25^. The A1 astrocytes express pro-inflammatory genes and the A2 astrocytes express anti-inflammatory genes. The pro-inflammatory A1 reactive astrocytes show upregulation of *Tnf* (Tumor Necrosis Factor), *Il-1b* (Interleukin 1-beta) and produce increased amounts of reactive oxygen species^51^. The anti-inflammatory A2 reactive astrocytes show upregulation of *Chil3* (Chitinase-like 3), *Fzd1* (Frizzled class receptor 1), and *Arg1*(Arginase 1)^52^.

Astrocytes become activated becoming reactive astrocytes in response to multiple types of stimuli including infections, ischemia, trauma, cerebrovascular insult, the autoimmune response as well as in response to pH or ion imbalances^53,54^. Activated astrocytes initiate the neuroinflammatory cascade *in vivo*, in part due to its response to the cytokine IL-1β^55^.

Experimental data in the literature reveal that astrocytes, activated by IL-1β *in vitro*, display the same biologic activity as the reactive astrocytes found in the brain lesions of patients with neurodegenerative changes such as Alzheimer’s disease, Multiple sclerosis, Parkinson’s disease, thus supporting their use as a model of CNS neuroinflammation in the *in vitro* assays^56–58^. Activated astrocytes proliferate and express cell surface adhesion molecules (CAMs) including the intercellular adhesion molecule (ICAM), the vascular cellular adhesion molecule (VCAM) and neuronal cellular adhesion molecule (NCAM)^59–61^. In the inflammatory state, soluble adhesion molecules (sCAMs) are often found circulating in the blood presumably after being shed from the activated vascular and immune cells^59,61^.

Activated glial cells generate significant amounts of ROS and reactive nitrogen species (RNS)^62,63^. Although these free radicals have been shown to be vital in the immunologic destruction of invading microbial pathogens, these ROS/RNS also cause significant oxidative stress and injury to neurons resulting in intracellular oxidative damage to proteins, DNA and lipids^63^. There are multiple natural antioxidants, found in vegetables, fruit and wine, which show free radical scavenging activity in chemical assays *in vitro*, such as the 2,2-diphenyl-1-picryl-hydrazyl-hydrate (DPPH) assay, but it is also vital to demonstrate that these compounds which demonstrate free radical scavenging activity *in vitro* also demonstrate biologic antioxidant activity *in vivo* confirming their capacity to protect cells against oxidative stress and injury in living organisms^64–67^.

Organic polyphenols will often downregulate pro-inflammatory mediators such as inducible Nitric Oxide Synthase (iNOS) and cyclooxygenase-2 (COX-2) via the reduction in the expression of Nuclear factor kappa-light-chain-enhancer of activated B cells (NF-κB) and Mitogen-activated Protein Kinase (MAPK)^65–67^. Some research papers have suggested that ingestion of food polyphenols may provide protection from neurodegenerative disorders^68–70^.

**Figure 1:**
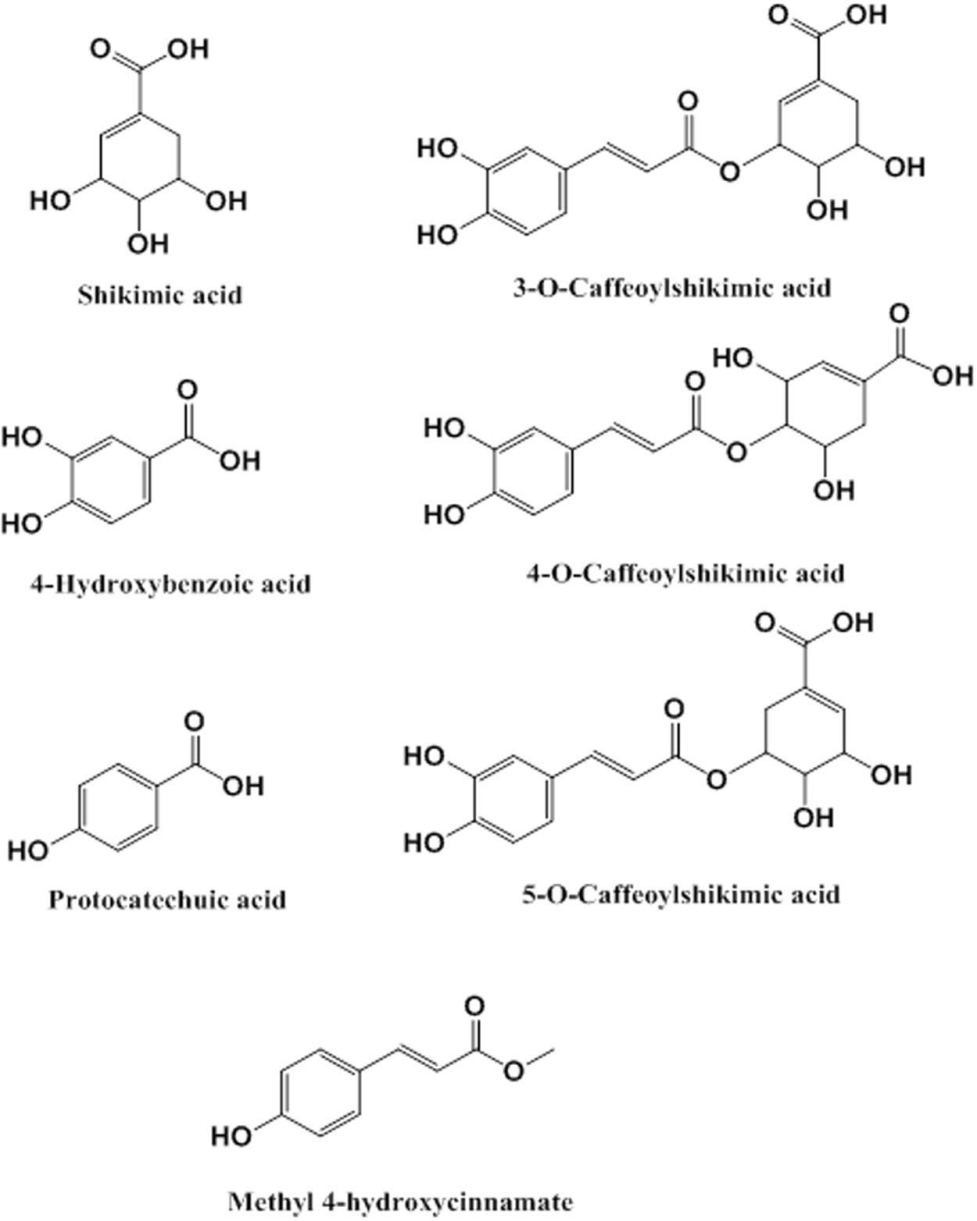
Some bioactive organic compounds in Palm Fruit Bioactives (PFB). PFB is a complex heterogeneous phytochemical mixture prepared from the aqueous extract of the oil palm fruit.

Palm Fruit Bioactives (“PFB”, also known in research literature as Oil Palm Phenolics (“OPP”)) represent a heterogeneous and complex aqueous mixture of water-soluble compounds derived from the fruit of the oil palm (*Elaeis guineensis*), extracted by the mechanical crushing, steaming and filtering processes as previously described^71,72^. PFB is rich in antioxidant polyphenols with multiple beneficial cellular and physiologic effects seen both *in vivo* and *in vitro* models^70–87^. The composition of PFB includes multiple organic compounds including protocatechuic acid, shikimic acid, *p*-hydroxybenzoic acid, methyl-4-hydroxycinnamate and three structural isomers of caffeoylshikimic acid as seen in Fig. 1^71^.

Multiple research studies show that PFB has diverse physiologic, cellular and biochemical effects which include: anti-inflammatory^76,77^, antidiabetic^78,79^, anticancer^80,81^, anti-angiogenic^82^, anti-hypertensive^83^, anti-atherosclerotic^84,85^, antiviral effects^86^, anti-chronic degenerative diseases of aging^82^, and anti-dementia by virtue of their inhibition of the aggregation of beta-amyloid peptide *in vitro*^87^. Toxicology studies show PFB to be very safe for oral consumption without any demonstrable toxic, carcinogenic or teratogenic effects^88^.

Much research suggests that antioxidants may modulate neuroinflammatory processes *in vitro*, so we decided to investigate the effects of PFB on reactive astrocytes *in vitro*. Prior studies by others show that PFB is a potent free radical scavenger and that PFB also upregulates cellular antioxidant gene pathways including Heme oxygenase (*HO-1*), cysteine reductase, glutathione synthetase^73,75,82^. In the neurodegenerative diseases, there are dysregulated genetic networks which involve an imbalance between the pro-inflammatory NF-κB pathways and the anti-inflammatory Nrf2 pathways. We have data which show that PFB reduces the NF-κB pathways in the network while it increases the Nrf2 pathways as revealed by RNA-seq (Koledova, Weinberg – unpublished data).

Other studies have shown that PFB and other polyphenols/flavonoids have anti-inflammatory properties by increasing the expression of inducible nitric oxide synthase (iNOS), cyclooxygenase and lipoxygenase, with focused effects on such cellular targets including macrophages, lymphocytes, endothelial and epithelial cells^89^. Multiple studies show the presence of chronic oxidative stress preceding several neurodegenerative diseases^12,62,63,69^. The current research suggests that chronic oxidative stress contributes to DNA damage, lipid peroxidation along with oxidative protein misfolding and aggregation, leading to progression of the disease. Current hypotheses suggest that the reduction of oxidative stress may ameliorate or slow the progression of neurodegenerative disease^90^.

Other studies show that PFB has neuroprotective effects in animal models^70,82^. The literature also shows that phenolic compounds and flavonoids, chemically similar to those found in PFB, have marked bioactive anti-inflammatory effects including such well-known antioxidants as curcumin and resveratrol^64,89,91^.

The typical neuroinflammatory processes result from interactions among various cytokines and chemokines secreted by immune cells, interacting with glial cells and neurons, causing a dysregulation of the CNS-immune-inflammatory network with upregulation of specific cell surface biomarkers such as cell adhesion molecules, and the production of reactive oxygen species (ROS) by glial cells, including astrocytes^52^. The details of the neuroinflammatory mechanism and interactions within the network of astrocytes-neurons and signaling pathways are still not fully understood.

In this study, we show that PFB has marked effects on astrocytes which have been activated by IL-1β. We measured specific biomarkers in the *in vitro* assays as surrogates for neuroinflammation. Our data show significant effects of PFB on: the secretion of cytokines and chemokines by the astrocytes, the production of ROS by the astrocytes and by changes in cell surface adhesion molecules expressed by the reactive astrocytes. The present study was undertaken to investigate how PFB may modulate the neuroinflammatory events associated with IL-1β-activated human astrocytes *in vivo*.

## Materials and Methods

### Palm Fruit Bioactives

PFB was prepared according to the methods described previously^72^. PFB is an aqueous extract derived from processed fruit of the African oil palm, *Elaeis guineensis*, which contains numerous bioactive phytochemical flavonoids and phenols. Among other compounds, PFB contains three isomers of caffeoylshikimic acid, protocatechuic acid, *p*-hydroxybenzoic acid, methyl-4-hydroxycinnamate and shikimic acid. The detailed composition of PFB has been previously published^72^. PFB can be prepared as a dried powder via a spray-drying process, in which form it is stored. In this experiment, a 9% aqueous PFB solution (weight/volume) was made from reconstituted spray-dried PFB extract. We then filtered the PFB solution through a 0.2-micron filter (Nalgene filter) prior to use in cell culture. The 9% aqueous PFB solution is equivalent to 2970 ppm Gallic Acid Equivalents (GAE) (as a measure of its reducing capacity in REDOX reactions).

Phenolic fractions of PFB were prepared via liquid-liquid extraction with ethyl acetate. Ethyl acetate and aqueous 9% PFB solution were well-mixed in a 50:50 mixture, then separated. The ethyl acetate fraction was dried and later reconstituted. The extraction was performed twice in succession. The resultant fractions are referred to as the phenolics fraction, partitioned in ethyl acetate, and non-phenolics fraction, partitioned in the aqueous phase. The phenolics fraction contains caffeoylshikimic acids, hydroxybenzoic acid, protacatechuic acid, and other relatively nonpolar compounds, while the nonphenolics fraction contains shikimic acid, soluble fibers, and other compounds. High-performance liquid chromatography was performed on each fraction to confirm separation of phenolic acids. The chromatograms at 280 nm may be found in Additional Fig. 2.

### Cell culture of normal human astrocytes

Normal human astrocytes (NHA) were obtained from Lonza Group (Basel, Switzerland, Cat. No. CC-2565) and regularly maintained in cell culture in our laboratory. The NHA were phenotypically characterized using anti-GFAP (anti-glial fibrillary acidic protein) and ~80% of the cells were GFAP-positive (Additional Fig. 1). The NHA were cultured in Astrocyte Growth Media (AGM, Lonza, Cat. No. CC-3186), supplemented with 3% fetal bovine serum (FBS), 1% L-glutamine, 0.25% insulin, and 0.1% ascorbic acid, gentamicin, and vascular endothelial growth factor (VEGF) and were incubated at 37 °C with 5% CO_2_. Before treatment, we synchronized cell cycle stages by supernatant exchange with serum-free medium for the following 24 hours. Afterward, medium exchange was performed with fresh AGM. At this time, to induce an inflammatory condition, cells were treated with recombinant human IL-1β (Sigma-Aldrich, St Louis, Missouri, USA) at a concentration of 20 ng/mL as commonly used for the activation of astrocytes^19,90,91^.

For the treated condition, PFB was added at concentrations of 0, 10, 20, or 40 µL/mL of the stock 9% PFB solution (which is equivalent to 0, 0.9, 1.8, 3.6 mg/mL) simultaneously to medium exchange and IL-1β treatment. In terms of antioxidant capacity, the PFB treatment concentrations may be expressed in terms of Gallic Acid Equivalents (GAE). The equivalent PFB treatment concentrations were 0, 30, 60 and 120 μg GAE/mL. The appropriate negative controls were also conducted, with no induction by IL-1β but with PFB treatment. Following a 24-hour or 96-hour incubation, an aliquot of the supernatant media was collected and stored at −20 °C for future assays (Luminex immunoassay). The cells were harvested, and proteins extracted, and stored at −20 °C for future assay.

### Western blotting analysis of Cell Surface Biomarkers

Following 24 hours treatment with IL-1β to activate the astrocytes, with or without PFB, astrocytes were washed twice with ice-cold PBS. Cellular protein was isolated from astrocytes which were lysed using ice-cold Radio-Immunoprecipitation assay (RIPA) buffer (Santa Cruz Biotechnology, California, USA). Protein concentrations were determined using Pierce BCA Protein Assay Kit from Pierce (Rockford, IL). Fifteen micrograms of protein lysate were loaded onto each lane and separated on a 7% polyacrylamide gel. Western blot analysis was performed with the following antibodies: mouse anti-ICAM (Santa Cruz Biotechnology, CA). The secondary antibodies were obtained from Rockland Immunochemicals Inc. (Limerick, PA). The blots were stripped and re-probed with mouse anti-β-actin (Santa Cruz Biotechnology, CA) to determine equivalent loading. The bands were detected on the Li-Cor Odyssey IR Infrared imaging system and analyzed by densitometry using the Li-Cor Image software. The Western blots were done in triplicate for each condition and specimen.

### Luminex cytokine and chemokine Multiplexing Immunoassay

Aliquots of cell supernatant at 24 or 96 hours were used for the Luminex bead-array multiplex immunoassay. We used the Milliplex Map Kit (Millipore, Billerica, Massachusetts USA). The Human Cytokine/Chemokines Magnetic Beat Panel (41-plex), catalogue number HCYTMAG-60K-PX41, was used to quantitatively measure cytokine and chemokine expression and secretion levels in the media collected from astrocytes treated with or without PFB in a range of concentrations in the presence or absence of IL-1β. The assay was performed according to the manufacturer’s instructions. This method quantifies soluble particles; in this case, molecules associated with inflammation using a fluorescence-based detection mechanism. The xPONENT 4.1 software was used for acquisition, while analysis was performed using MILLIPLEX Analyst 5.1 (Millipore, USA).

### DCFDA Assay for Intracellular Reactive Oxygen Species (ROS)

To measure the level of oxidative stress within cells, the reactive oxygen species-sensitive probe 5- and 6-chloromethyl-2′7′-dichlorofluorescin diacetate (DCFDA) was used (Abcam, Cambridge, Massachusetts, USA, Cat. No. ab113851). DCFDA is a cell-permeable, fluorogenic dye that measures hydroxyl, peroxyl and other reactive oxygen species (ROS) activity within the cell. The fluorescence intensity of intracellular DCFDA is a linear indicator of amount of ROS activity in the cells. To detect the accumulation of ROS, astrocyte cultures were incubated with DCFDA at 5 µM for 30 min at 37 °C, washed twice with PBS. After that, astrocytes were treated either with IL-1β at 20 ng/mL alone or with a range of PFB concentrations in the presence or absence of IL-1β for a 24-hour period. The ROS level was measured as a function of the relative fluorescence via excitation and emission spectroscopy at wavelengths of 485 and 535 nm respectively.

### Statistical Analysis of the Results

The experiments using cultured astrocytes were performed a minimum of three times. The values are expressed as the mean ± standard error of the mean (SEM). Statistical analysis was performed using the Student’s t-test. Results were considered statistically significant if the p-value obtained from the Student’s t-test reached a significance level of p < 0.05.

### Ethics Approval and Consent to Participate

Mr. Michael J. Keohane of the M.I.T. Committee on the Use of Humans as Experimental Subjects (COUHES) [http://couhes.mit.edu/] has stated that COUHES review is not required for commercially available, de-identified samples which applies to the Normal Human Astrocytes which we purchased from Lonza, Basel, Switzerland. Thus there was no need to obtain any review or approval of these cell lines which were purchased from Lonza.

## Results

### Basal cytokine/chemokine profile of human astrocytes

We initially established the basal cytokine/chemokine profile of unstimulated human astrocytes. The basal expression of 37 cytokines/chemokines secreted by the cultured astrocytes incubated without cytokine IL-1β (unstimulated) were measured at two time points: 24 hours (Table 1) and 96 hours (Table 2). At 24 hours, we were able to detect 20 out of 37 cytokines and chemokines: eotaxin, fractalkine, G-CSF, GM-CSF, GRO, IFNα2, IFNγ, IL-6, IL-8, IL-12 (p40), IP-10, MCP-3, MDC, MIP-1α, MIP-1β, PDGF-AA, RANTES, ScD40L, TGFα, TNFα. At 96 hours, 5 cytokines/chemokines were detected (FGF-2, IL-1α, IL-7, IL-12(p70), PDGF-AB/BB) in addition to the same 20 cytokines/chemokines detected at 24 hours.

**Table 1:** Cytokines/chemokines secreted by human astrocytes at 24 hours with/without IL-1β activation.

Protein	Gene location that encodes protein	24 h protein concentration Avg ± 1 STD (pg/mL)	24 h IL-1β stimulation fold change (ratio)
G-CSF	17q21.1	10.4 ± 7.7	**570**.1*
GM-CSF	5q31.1	4.5 ± 1.8	**574**.6*
RANTES	17q12	4.0 ± 0	**395**.4^***^
GRO	4q13.3	78.8 ± 40.9	**197**.5*
IL-8	4q13.3	147.6 ± 72.6	**97**.9***
TNFα	6p21.33	3.2 ± 0	**46**.5*
IL-6	7p15.3	579.2 ± 312.4	**23**.2*
MIP-1β	17q12	3.9 ± 0	**22**.5*
IP-10	4q21.1	28.0 ± 17.0	**20**.1*
Eotaxin	17q12	4.5 ± 0.0	**7**.4*
MIP-1α	17q12	27.1 ± 0.5	5
MCP-3	17q12	20.1 ± 2.2	3.1
IFNα2	9p21.3	9.1 ± 2.7	4.6
Fractalkine	16q21	54.1 ± 17.2	**2**.5*
IFNγ	12q15	4.4 ± 2.2	2.4
IL-12(p40)	5q33.3	6.2 ± 3.6	1.7
TGFα	2p13.3	40.4 ± 30.3	1.3
MDC	16q21	4.8 ± 2.5	1.3
sCD40L	Xq26.3	8.2 ± 3.3	1.1
PDGF-AA	7p22.3	155.5 ± 7.5	0.8
IL-12(p70)	3Q25.33 AND 5q33.3	<3.2	Abs 10.4 ± 5.2
IL-1α	2q14.1	<2.7	Abs 7.3 ± 1.6
IL-7	8q21.13	<3.2	Abs 24.8 ± 17.9
PDGF-AB/BB	7p22.3 & 22q13.1 or 22q13.1 & 22q13.1	<22.0	Abs 24.4 ± 18.8
FGF-2	4q28.1	<40.5	Abs 57.5 ± 20.3
Flt3L	19q13.33	ND	ND
IL-2	4q27	ND	ND
IL-3	5q31.1	ND	ND
IL-5	5q31.1	ND	ND
IL-9	5q31.1	ND	ND
IL-10	1q32.1	ND	ND

For each cytokine, values are the mean concentration +/− standard deviation for the unstimulated condition and as fold-change ratios for the IL-1β-stimulated condition. The inflammatory profile (bolded text) consists of the secreted cytokines/chemokines altered in production with IL-1β stimulation as compared to their basal expression levels. Student’s t-test using absolute values, *p < 0.05, **p < 0.01, ***p < 0.001. Experiments were performed in triplicate.

**Table 2:** Cytokines/chemokines secreted by human astrocytes at 96 hours with/without IL-1β activation.

Protein	Gene location that encodes protein	96 h protein concentration Avg ± 1 STD (pg/mL)	96 h IL-1β stimulation fold change (ratio)
RANTES	17q12	22.2 ± 12.6	**227**.7**
IP-10	4q21.1	34.0 ± 20.7	**80**.8*
GM-CSF	5q31.1	253.1 ± 376.1	**27**.3*
GRO	4q13.3	699.6 ± 486.3	**21**.1^*^
G-CSF	17q21.1	463.1 ± 561.2	23.7
MIP-1α	17q12	24.4 ± 5.0	20.5
TNFα	6p21.33	18.0 ± 17.5	**10**.2*
IL-8	4q13.3	1581.5 ± 1289.7	**7**.**2**
MCP-3	17q12	82.6 ± 79.7	**6**.4**
MIP-1β	17q12	18.2 ± 8.8	**5**.2*
IL-6	7p15.3	3980.1 ± 3442.9	**4**.0*
IL-1α	2q14.1	8.2 ± 1.3	3.6
IFNα2	9p21.3	23.3 ± 9.4	**2**.4*
sCD40L	Xq26.3	5.9 ± 2.6	2.1
Fractalkine	16q21	101.1 ± 49.1	2**
TGFα	2p13.3	63.6 ± 43.1	1.9
Eotaxin	17q12	31.1 ± 28.8	1.5
IL-12(p40)	5q33.3	8.8 ± 3.1	1.4
IL-12(p70)	3Q25.33 AND 5q33.3	6.4 ± 0.3	1.4
PDGF-AB/BB	7p22.3 & 22q13.1 or 22q13.1 & 22q13.1	157.7 ± 110.0	1.4
IFNγ	12q15	5.7 ± 2.0	1.3
MDC	16q21	5.4 ± 1.6	1.2
PDGF-AA	7p22.3	2183.2 ± 2002.0	1.2
IL-7	8q21.13	29.5 ± 3.5	1.1
FGF-2	4q28.1	68.6 ± 26.5	1.1
Flt3L	19q13.33	ND	ND
IL-2	4q27	ND	ND
IL-3	5q31.1	ND	ND
IL-5	5q31.1	ND	ND
IL-9	5q31.1	ND	ND
IL-10	1q32.1	ND	ND

Values are given as average concentrations +/− standard deviation for the unstimulated condition and as fold-change ratios for the IL-1β-stimulated condition. The inflammatory profile (bolded text) consists of the secreted cytokines/chemokines with significant changes upon IL-1β stimulation as compared with their basal expression levels as based on significant difference. Student’s t-test using absolute values, *p < 0.05, **p < 0.01, ***p < 0.001.

At 24 hours, of the 20 detected cytokines/chemokines, 10 analytes were found in concentrations less than 10 pg/mL (eotaxin, GM-CSF, IFNα2, IFNγ, IL-12 (p40), MDC, MIP-1β, RANTES, sCD40L, TNFα). There were 7 analytes between concentrations 10 and 100 pg/mL (fractalkine, G-CSF, GRO, IP-10, MCP-3, MIP-1α, TGFα), and 2 analytes were found in concentrations between 100 and 500 pg/mL (IL-8, PDGF-AA), and 1 analyte was above concentration 500 pg/mL (IL-6) (Table 1).

At 96 hours, of the 25 detectable cytokines and chemokines, 6 analytes were found in concentrations less than 10 pg/mL (IL-1α, sCD40L, IFNγ, IL-12 (p40), IL-12 (p70), MDC), 11 analytes were in concentration range 10 to 100 pg/mL (Eotaxin, FGF-2, IFNα2, IL-7, IP-10, MCP-3, MIP-1α, MIP-1β, RANTES, TGFα,TNFα), 4 analytes were detected in concentrations between 100 and 500 pg/mL (Fractalkine, G-CSF, GM-CSF, PDGF-AB/BB), and 4 analytes were found in concentrations above 500 pg/mL (GRO, IL-6, IL-8, PDGF-AA) (Table 2). More individual cytokines/chemokines were detected at 96 h than 24 h, indicating increased levels of expression.

### Cytokine profile of human astrocytes with IL-1β stimulation

Following 24-hour exposure to IL-1β, 11 cytokines/chemokines were significantly increased compared to controls (eotaxin, fractalkine, G-CSF, GM-CSF, GRO, IL-6, IL-8, IP-10, MIP-1β, RANTES, TNFα). In Table 1, the analytes that were significantly changed by IL-1β stimulation at 24 h, along with analytes present but not significantly changed by IL-1β stimulation, are shown based on fold change. The absolute cytokine/chemokine concentrations are located in Additional Table 1.

Following 96-hour exposure to IL-1β, 10 cytokines showed significantly increased expression: fractalkine, GM-CSF, GRO, IFNα2, IL-6, IP-10, MCP-3, MIP-1β, RANTES, and TNFα (Table 2). There was no correlation between the basal levels of cytokine expression in unstimulated astrocytes compared with the IL-1β-stimulated levels of cytokine expression.

There were 6 cytokines/chemokines that were not detectable in the basal unstimulated condition but were only detectable after IL-1β stimulation. These include IL-13, IL-15, IL-17a, IL-1RA, IL-4, TNFβ (Table 3). Many of these cytokines/chemokines had concentrations less than 10 pg/mL (including IL-12 (p70), IL-13, IL-15 IL-17a, TNFβ). IL-4 had a concentration in the range 10 to 100 pg/mL (Table 3). Several cytokines/chemokines were not detected in any condition (FLT3L, IL-2, IL-3, IL-5, IL-9, IL-10) (Tables 1 and 2).

**Table 3:** Cytokines/chemokines which are secreted by human astrocytes only upon IL-1β activation.

Protein	Gene location that encodes protein	24 h protein concentration with IL-1β stimulation Avg ± 1 STD (pg/mL)	96 h protein concentration with IL-1β stimulation Avg ± 1 STD (pg/mL)
IL-4	5q31.1	Abs 15.6 ± 2.0	Abs 21.9 ± 8.1
IL-13	5q31.1	Abs 5.4 ± 0.8	Abs 7.1 ± 0.3
IL-15	4q31.21	Abs 3.9 ± 0.7	Abs 11.5 ± 5.2
IL-17A	6p12.2	Abs 4.5 ± 2.1	Abs 5.1 ± 1.9
IL-1RA	2q14.1	Abs 17.9 ± 0.9	Abs 14.5 ± 14.5
TNFβ	6p21.33	Abs 4.5 ± 1.8	Abs 4.7 ± 2.2

Cytokine concentrations are the average concentration +/− standard deviation. These cytokines were only detectable upon IL-1β-stimulation, and we consider them to be part of the inflammatory profile.

**Figure 2:**
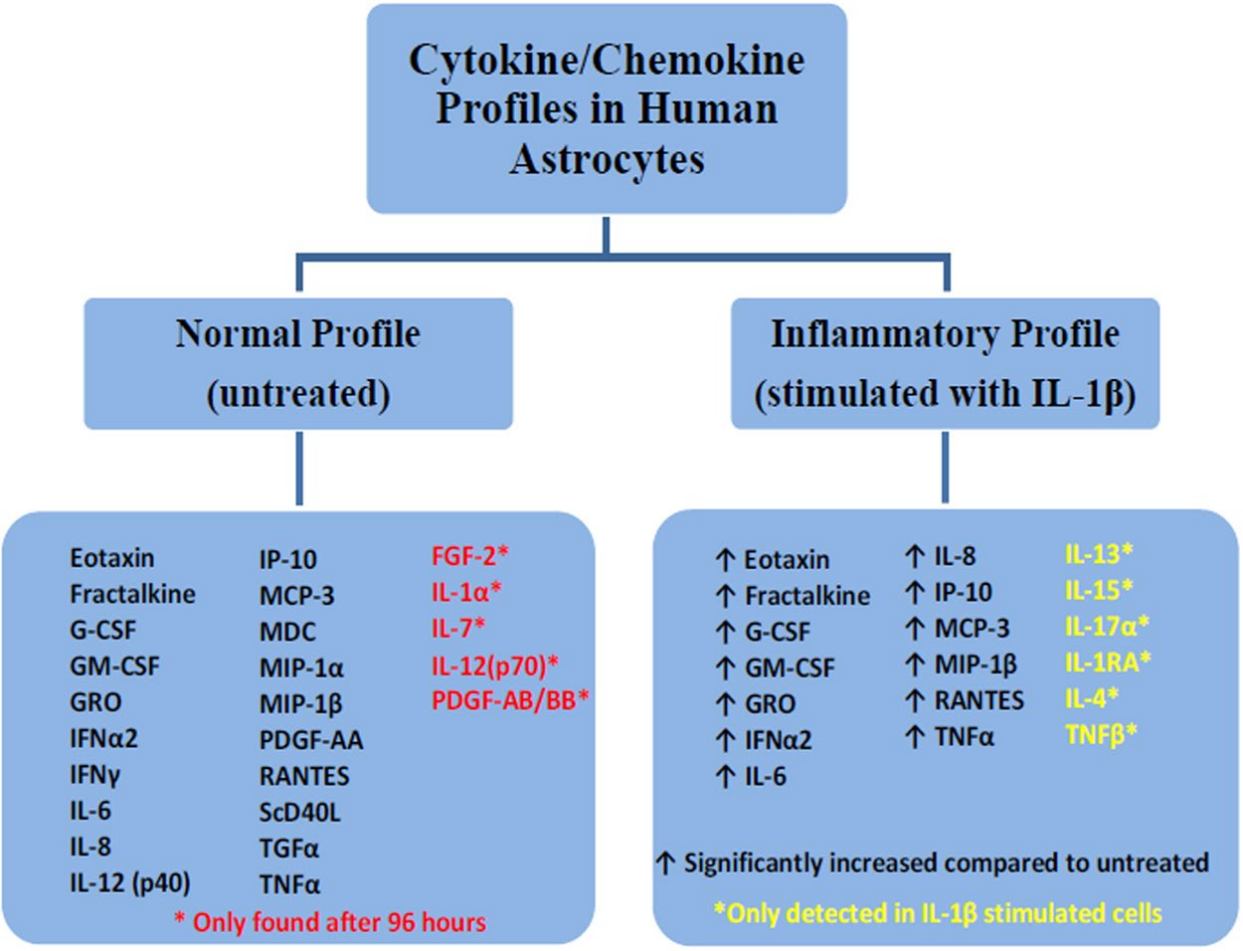
Profiles of cytokines/chemokines produced by human astrocytes with/without IL-1β stimulation reveal 2 profiles: normal (basal) and inflammatory (post-IL-1β) The inflammatory profile consists of those secreted cytokines/chemokines which were significantly altered with IL-1β stimulation compared to their basal expression levels at 24 h or 96 h, while the normal profile consists of those cytokines/chemokines detectable in the basal state.

When comparing the unstimulated NHA with the IL-1β-stimulated NHA, distinct subsets of the cytokines and chemokines became apparent. There appear to be two distinct profiles of cytokine/chemokine production. We define the normal profile of cytokines/chemokines as those detected in the untreated condition which are not significantly changed upon stimulation with IL-1β. We define the inflammatory profile of cytokines/chemokines to include those which are significantly increased upon IL-1β stimulation as well as those which are not detected in unstimulated NHA but are detected upon IL-1β stimulation (Fig. 2).

**Figure 3:**
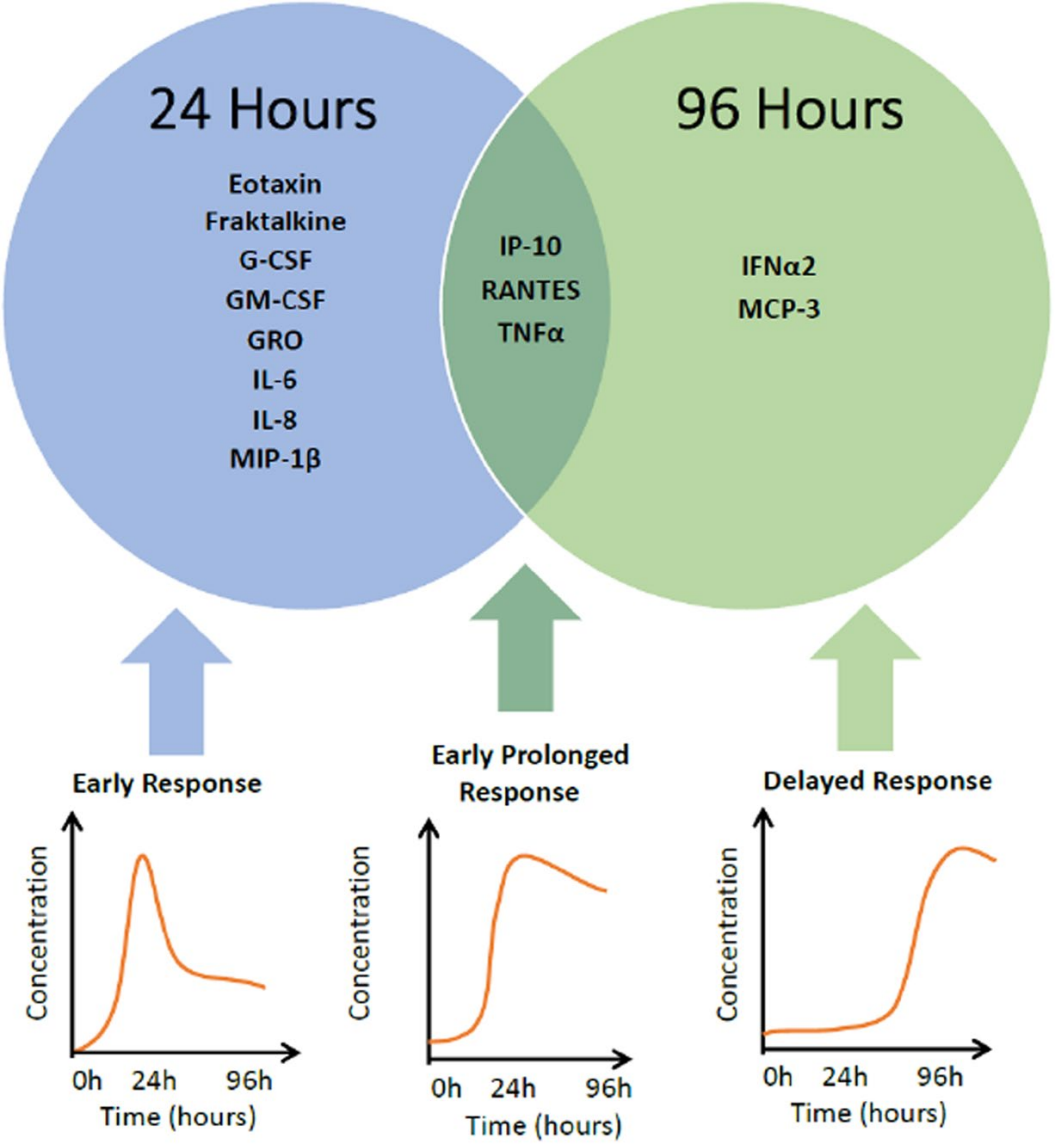
Time-dependent differential expression of cytokines/chemokines induced by IL-1β stimulation in human astrocytes. Cytokines induced within 24 hours are designated “early short response.” Cytokines produced after 96 hours of induction are designated “delayed response.” Cytokines induced within 24 h which remain elevated at 96 hours are designated “early-prolonged response”.

When comparing the temporal dynamics of the 24-hour levels and the 96-hour levels of the IL-1β-stimulated groups, the cytokines and chemokines fall into three distinct groups (Fig. 3). The first group represents those cytokines/chemokines which are markedly increased during the first 24-hour period following IL-1β stimulation and then quickly decrease by the 96-hour measurement. These include eotaxin, fractalkine, G-CSF, GM-CSF, GRO, IL-6, IL-8, MIP-1β. We call this first group the early-short response group.

The second group of cytokines/chemokines remain near basal levels for the first 24-hours post-IL-1β stimulation but then show a marked increase from the 24-hour time measurement to the 96-hour measurement. These include IFNα2 and MCP-3. We call this group the strong-delayed response group.

Finally there is the third type of cytokines/chemokines which show marked increase during the first 24 hours post–IL-1β stimulation and remain high through the 96-hour time measurement. These cytokines/chemokines include IP-10, RANTES and TNFα. We call this group the early-prolonged group.

### Effect of PFB on the basal cytokine profile of human astrocytes

PFB has no significant effect on the cytokine/chemokine profile of astrocytes which are not activated by IL-1β. Data is shown in Additional Table 2 (24 hours) and Additional Table 3 (96 hours).

### Effect of PFB on the profile of IL-1β-stimulated human astrocytes

PFB has a marked effect on the production of cytokines/chemokines which belong to the inflammatory profile, i.e. the group of 13 cytokines/chemokines which were significantly elevated in astrocytes stimulated with IL-1β. A significant decrease is seen in the levels of 3 cytokines/chemokines in the inflammatory profile after 24 hours of treatment with PFB: RANTES, IP-10 and TNFα. An even greater decrease was observed in the levels at 96 hours. These results were confirmed three times on subsequent passages of NHA, in duplicate for each passage. The individual values for each passage are listed in Additional Table 4. The different passages of NHA expressed varying levels of cytokine/chemokine concentration. All passages of NHA possessed the exact same tendency to decrease production of RANTES, IP-10, and TNFα in response to PFB treatment. We note that these cytokines/chemokines belong to the early and prolonged response group mentioned previously (Fig. 3).

**Figure 4:**
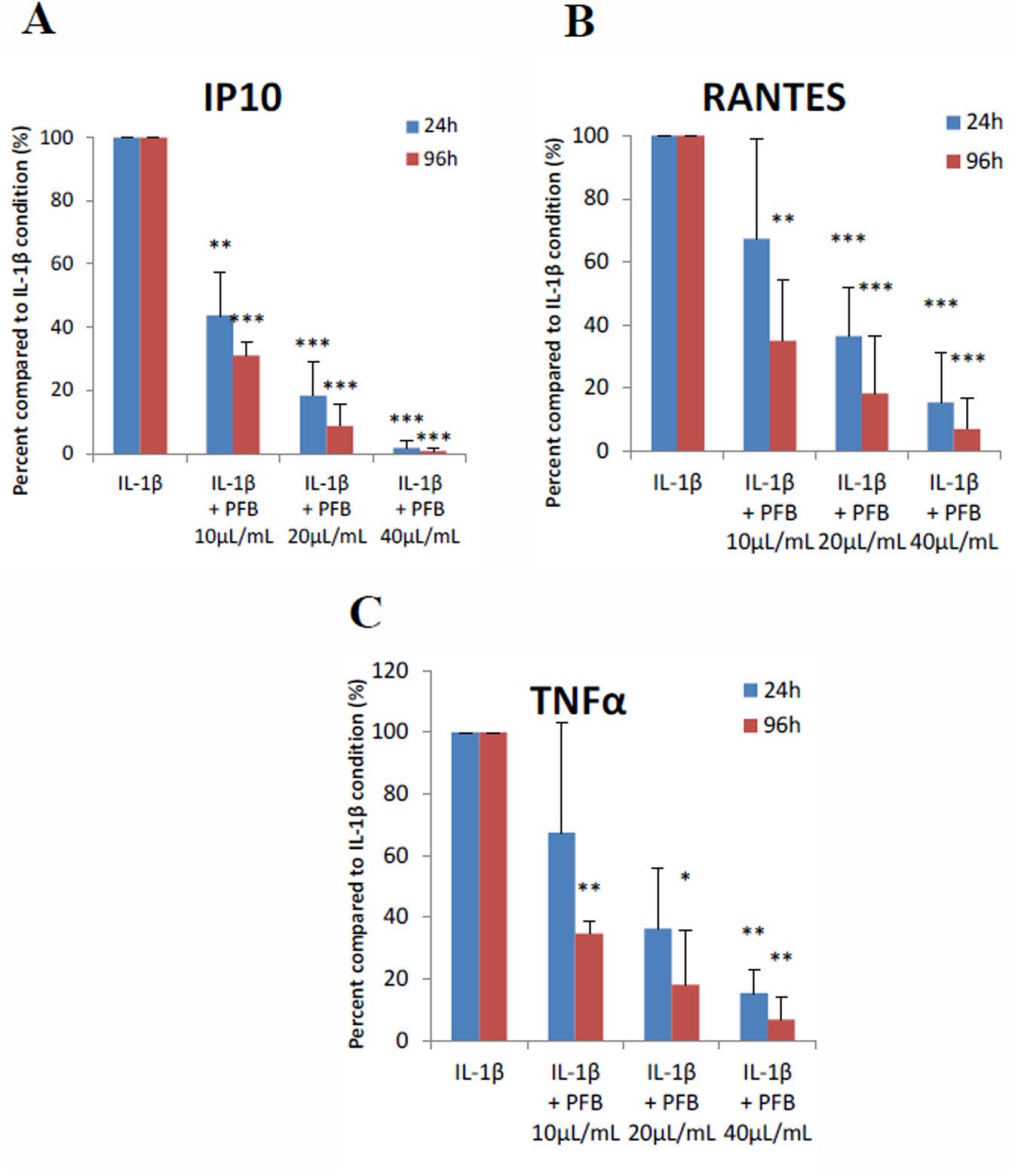
(**A**) IP-10, (**B**) RANTES, and (**C**) TNFα secretion levels induced in NHA by IL-1β stimulation (20 ng/ml) are significantly reduced with exposure to Palm Fruit Bioactives at 24 and 96 hours in a dose-dependent manner. The normalized percent change (%) for each PFB treatment condition compared with the positive control, IL-1β stimulation alone, for each time point. Data represent the mean percentage change ± SD of three Luminex replicates (*p < 0.05, **p < 0.01, ***p < 0.001).

To summarize the results of our analysis, we calculated the mean percent change. Figure 4 shows that in NHA stimulated by IL-1β, a significant decrease in production of RANTES, IP-10, and TNFα after 24 hours and an even greater decrease in production at 96 hours occurred in the presence of PFB in a dose-dependent manner (absolute cytokine/chemokine levels may be seen in Additional Table 4). With PFB exposure, IP-10 expression was reduced to levels equivalent to normal, non-stimulated NHA; a similar effect was observed in expression levels of RANTES and TNFα: exposure to PFB markedly decreased their expression in IL-1β-stimulated NHA, reducing them toward normal basal levels. In addition, a reduction in the expression of MCP-3, GM-CSF, G-CSF and MIP-1β was observed, but this was not a statistically significant.

### Reduction of IP-10 by PFB exposure in IL-1β-stimulated NHA

These results show that in IL-1β-stimulated NHA, IP-10 expression was decreased by 56% and 69% at 24 and 96 hours exposure, respectively, in the presence of PFB at a concentration of 10 μL/mL; an even greater decrease was observed at higher concentrations. At a PFB concentration of 20 μL/mL, IP-10 was decreased by 81% and 91% at 24 and 96 hours, respectively, and at a PFB concentration of 40 μL/mL, IP-10 was decreased by 98% and 99% respectively, restoring IP-10 toward the normal basal levels for NHA (Fig. 4a).

### Reduction of RANTES by PFB exposure in IL-1β-stimulated NHA

These results show that in IL-1β-stimulated NHA, RANTES expression was decreased by 65% at 96 hours post-exposure to PFB at a concentration of 10 μL/mL; the reduction in RANTES was not significant at 24 hours (Fig. 4b). A greater reduction in RANTES expression was observed at higher concentrations of PFB exposure. At a PFB concentration of 20 μL/mL, RANTES was decreased by 64% and 82% at 24 and 96 hours, respectively, and for a PFB concentration of 40 μL/mL, RANTES was decreased by 85% and 93% respectively. Exposure to PFB greatly reduced RANTES levels in IL-1β-stimulated NHA.

### Reduction of TNFα by PFB exposure in IL-1β-stimulated NHA

These results also show that TNFα expression was decreased in IL-1β-stimulated NHA with PFB exposure. At 24 hours of exposure, we observed a tendency toward lower expression at PFB concentrations of 10 μL/mL and 20 μL/mL, but these differences were not statistically significant. There was a significant reduction in TNFα expression when in the presence of 40 μL/mL of PFB. Exposure to PFB 40 μL/mL decreased TNFα production by 81% at 24 hours (Fig. 4c). At 96 hours of exposure, TNFα expression was decreased by 57% with PFB at a concentration of 10 μL/mL. A greater decrease in TNFα expression was observed at higher concentrations of PFB. At a PFB concentration of 20 μL/mL, TNFα was decreased by 66% and at a PFB concentration of 40 μL/mL, TNFα was decreased by 94%. Exposure to PFB greatly reduced TNFα levels in IL-1β-stimulated NHA, by the 96 hour time measurement.

#### Comparison of phenolic with non-phenolic fractions of PFB on IP10, RANTES and TNFα

**Figure 5:**
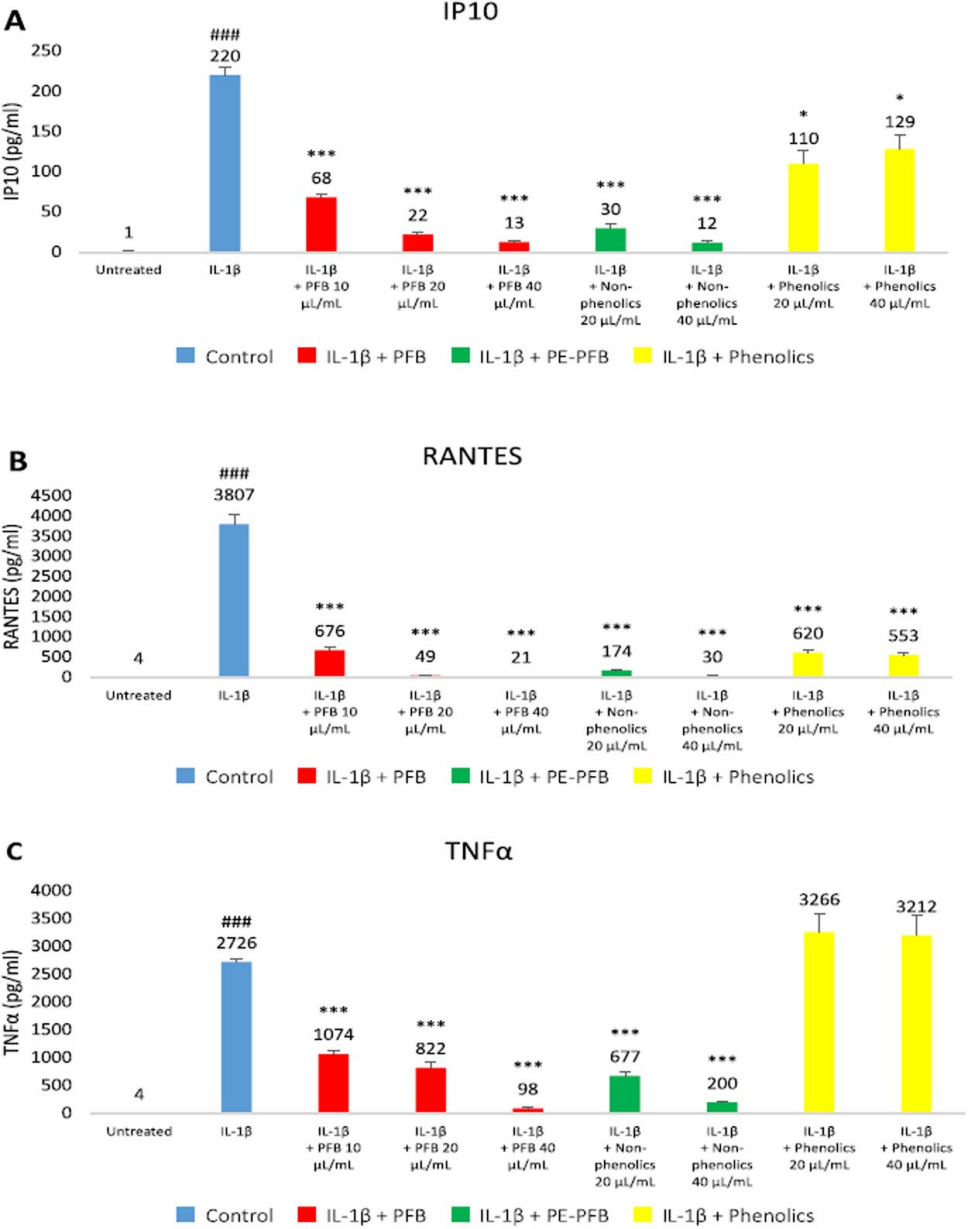
Effects of phenolic vs. non-phenolic fractions of PFB on (**A**) IP10, (**B**) RANTES, and (**C**) TNFα levels in IL-1β-stimulated NHA. Concentrations of IP10, RANTES, TNFα for untreated and IL-1β stimulated controls, and IL-1β for different treatment conditions: PFB, non-phenolic PFB fraction, and phenolic PFB fraction at 20 and 40 μL/mL concentrations for each condition. Mean and standard deviation given for three replicates. Student’s t-test, ^###^p < 0.001, untreated vs. IL-1β stimulation; ***p < 0.001, IL-1β vs. IL-1β + PFB or PFB fraction.

Several of the organic compounds in PFB can be classified as either phenolic or non-phenolic. We wanted to compare the relative contributions of phenolic compounds versus non-phenolics, so we fractionated the PFB. Additional Fig. 2 shows the chromatograms for the phenolic fraction and the non-phenolic fraction. After determining that PFB shows a dose-dependent reduction of IP10, RANTES and TNFα, we tested the relative potency of the PFB fractions including the phenolic and non-phenolic fractions. In Fig. 5, we see that the non-phenolic fraction shows similar potency as the unfractionated PFB. The phenolic fraction shows no significant effect on TNFα, moderate effect on IP10 reduction, and the greatest effect on RANTES reduction, but still less than the unfractionated PFB. This suggests that other non-phenolic compounds play a greater role in this cytokine/chemokine reduction than the phenolic fraction of PFB.

### Effect of PFB on production of ROS by IL-1β-stimulated human astrocytes

**Figure 6:**
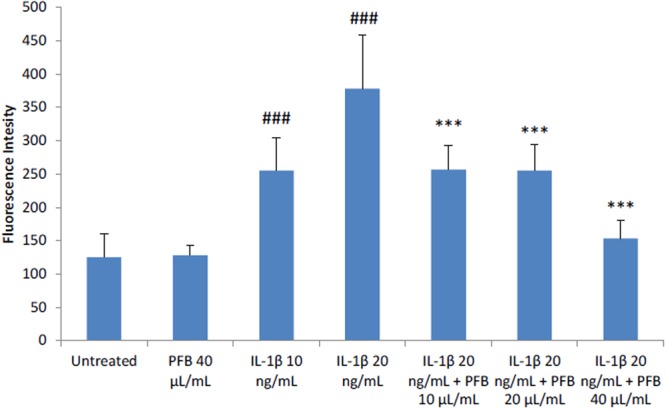
Elevated Reactive Oxygen Species production in NHA stimulated by IL-1β is reduced by treatment with Palm Fruit Bioactives, as measured by DCFDA Assay after 24 h. No change is observed between ROS production between untreated NHA and the highest concentration of PFB treatment used, while increased ROS production from stimulation is decreased by PFB. Fluorescence intensity correlates with level of oxidative stress per condition in the DCDFA Assay. Values given as mean fluorescence intensity +/− standard deviation of three replicates. Student’s t-test, ^###^p < 0.001, untreated control vs. IL-1β; ***p < 0.001, IL-1β vs. PFB-X + IL-1β, where X is treatment concentration of PFB.

The production of ROS by NHA was measured by a DCDFA assay in seven experimental groups: unstimulated, unstimulated treated with PFB (40 μL/mL), stimulated with IL-1β (10, 20 ng/mL), and stimulated with IL-1β (20 ng/mL) with PFB treatment (10, 20, 40 μL/mL). 24-hour exposure of PFB alone has no effect on ROS production by astrocytes. IL-1β causes a dose-dependent increase in ROS production (Fig. 6).

Interestingly, when PFB was present in combination with IL-1β, a marked decrease in ROS production was observed as compared with the NHA treated only with IL-1β (Fig. 6). PFB treatment in concentrations of 10 and 20 μL/mL had approximately the same effect on ROS production in IL-1β (20 ng/mL) stimulated NHA. In both cases, the production of ROS was reduced toward the level of ROS measured in NHA treated with IL-1β at a concentration of 10 ng/mL, and these levels were lower than the the levels of ROS measured in NHA treated with IL-1β at a concentration of 20 ng/mL. The greatest decrease in ROS production in IL-1β-treated NHA (20 ng/mL) was observed at the highest treatment concentration of PFB (40 μL/mL); ROS levels were observed to be close to the level seen in untreated cells. Overall, PFB reduced ROS production in IL-1β stimulated NHA.

### Effect of PFB on Astrocyte Cell Adhesion Molecules

**Figure 7:**
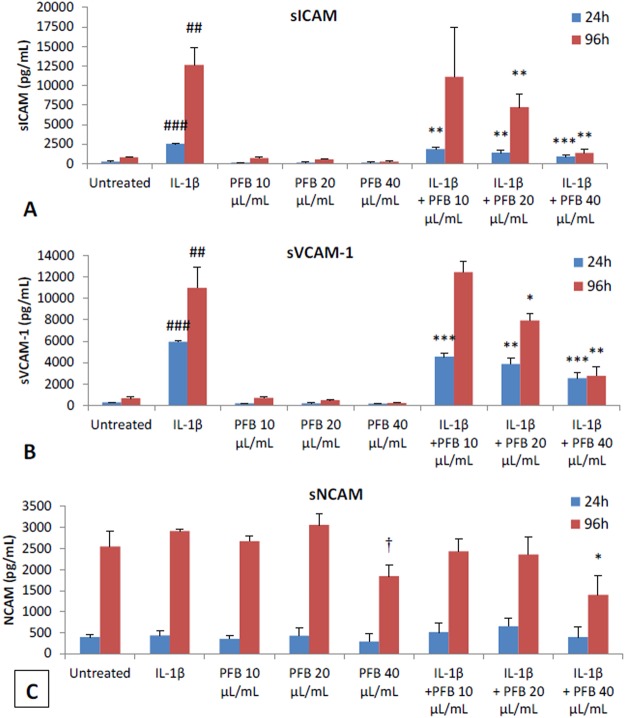
Effect of PFB on soluble adhesion molecules expressed by NHA with 24 h or 96 h stimulation with IL-1β. (**A**,**B**) PFB exposure decreased siCAM and sVCAM expression in both unstimulated and IL-1β-stimulated NHA. (**C**) NCAM expression did not differ upon exposure to IL-1β. Values given as cytokine concentration, mean +/− SD. Experiment was performed in triplicate. ^##^p < 0.01, ^###^p < 0.001, untreated control vs. IL-1β; *p < 0.05, **p < 0.01, ***p < 0.001, IL-1β vs. PFB-X + IL-1β.

After NHA were activated by incubation with IL-1β for either 24 or 96 hours and subsequently were treated with a range of PFB concentrations (0, 10, 20, 40 μL/mL), the expression of cell adhesion molecules ICAM, sICAM, sVCAM, and sNCAM was measured. sICAM, sVCAM, and sNCAM expression was measured by Luminex immunoassay (Fig. 7).

### Effect of PFB on soluble adhesion molecules (sICAM, sVCAM, sNCAM)

The astrocytes also shed a soluble form of ICAM (sICAM) as well as soluble adhesion molecules sVCAM and sNCAM into the culture supernatant. These soluble adhesion molecules were measured using the multiplex Luminex immunoassay magnetic bead array assay. The results show that PFB causes a statistically significant reduction in the levels of of sICAM and sVCAM at 24 hours with an even greater reduction at 96 hours (Fig. 7). There was no significant effect with IL-1β stimulation in expression of sNCAM.

There is a dose-dependent modulation of the expression of sICAM and sVCAM seen at 24 hours with PFB treatment. This modulation by PFB treatment is even greater at 96 hours. There were no marked effects of PFB treatment on expression of sNCAM. The greatest decrease in expression of sICAM and sVCAM occurred with the highest PFB treatment concentration (40 μL/mL).

IL-1β-activated human astrocytes show marked upregulation of multiple biomarkers and with the release of multiple inflammatory mediators. We show significant inhibition of these functions by simultaneously co-incubating the astrocytes with PFB and IL-1β. PFB causes a dose-dependent and time-dependent reduction in specific cytokines TNFα, RANTES, and IP-10.

Our results also show that PFB significantly reduces ROS production by IL-1β-activated astrocytes. Activated astrocytes normally express increased levels of cell adhesion molecules. PFB reduces the expression of ICAM and VCAM, both in activated and naïve human astrocytes *in vitro*.

## Discussion

Recent studies have focused on the important role of dysfunctional astrogliosis in neurologic disease^12,15,17,19,92,93^. These studies have pointed to the importance of activated astrocytes as a therapeutic target. Astrocytes play a key role in signaling in both the innate and adaptive immune responses^12,20,22,23^. A key component of this signaling process involves the production and release of cytokines, chemokines and other inflammatory mediators including reactive oxygen species^19,52,53,57,92,93^. Physiologically these signaling pathways play a protective role in fighting CNS infections and tumors^19,92^. However, when excessive, chronic or dysregulated, these signaling mediators may initiate a dysregulated, pathologic or autoimmune response^19,93–105^. These dysregulated responses cause significant neuronal injury and death and may lead to neurodegenerative disorders.

Based on prior data showing that PFB reduces the expression of NF-κB^80^, we decided to investigate the effects of PFB on cytokine/chemokine secretion by IL-1β-activated astrocytes to explore the immunomodulatory and anti-inflammatory effects under these conditions. Our data show that there are several distinct subsets of cytokine/chemokine profiles based on temporal expression and responsiveness to IL-1β. We identified the basal profile as those cytokines/chemokines expressed by unstimulated NHA in the basal state and the inflammatory profile as those cytokines/chemokines which are expressed *de novo* after stimulation with IL-1β as well as those which are significantly upregulated following IL-1β stimulation (Fig. 2).

The inflammatory profile of cytokines/chemokines demonstrates distinct patterns of temporal expression with IL-1β incubation at the 24-hour and 96-hour time points. The kinetics for the expression of the cytokines/chemokines reveal 3 distinct groups: (1) the early-short response, (2) the delayed response and (3) the early-prolonged response. The early-short response cytokines reach maximum expression at 24 hours and then decrease; these include eotaxin, fractalkine, G-CSF, GM-CSF, GRO, IL-6, IL-8, and MIP-1β. The delayed response cytokines reach maximum expression at 96 hours, and include MCP-3 and IFNα2. The early-prolonged response cytokines reach maximum response at 24 hours and remain elevated at 96 hours. These include IP-10, RANTES, and TNFα.

These results show that PFB has no significant effect on the early-short response or the delayed-response cytokines. However, we do observe slight reductions in the expression of G-CSF, GM-CSF, MCP-3, and MIP-1β, but these are not statistically significant. The most prominent cytokine effect of PFB is its significant reduction of the expression of the early-prolonged cytokines IP-10, RANTES, and TNFα in a dose-dependent manner. These cytokines have been demonstrated to play a crucial role in such neurodegenerative diseases. It is well-established that IP-10, RANTES, and TNFα are significantly increased in Alzheimer’s disease (AD), Parkinson’s disease (PD), Multiple Sclerosis (MS), HIV-associated dementia and Neuropathic Pain Syndrome^96–115^.

TNFα plays a key role in AD pathogenesis^114^. TNFα induces significant production of inducible nitric oxide synthase (iNOS), neuronal stress and dysfunction leading to neuronal death^115^. It is well established that activated astrocytes synthesize and release TNFα, which stimulates increased expression of amyloid precursor protein (APP) by glial cells^116–118^, and also upregulates the conversion of APP into the pathologic variants of beta-amyloid^119–121^. The current belief is that reducing the level of TNFα may be beneficial in neuro-inflammatory conditions. As our results show that PFB reduces the secretion of TNFα by activated astrocytes, we expect that it may have a beneficial action in pathologies such as Alzheimer’s, where TNFα plays a key role in early dysfunction.

Elevated levels of TNFα are found in the brain and CSF of patients with Parkinson’s disease (PD) and mouse models of PD^107,122^. The importance of TNFα-induced signaling in the pathogenesis of PD is corroborated in multiple animal models of PD including Parkinsonian syndrome induced by the neurotoxins MPTP (1-methyl-4-phenyl-1,2,3,6-tetrahydropyridine) and rotenone, which simulate the TNFα-signaling seen clinically in patients with PD^123–125^. In a mouse model of amyotrophic lateral sclerosis (ALS), the progression of neurodegeneration is accompanied by elevation of TNFα mRNA transcripts and protein in the reactive microglia found in the spinal cord^105,111,126^. Relapses of multiple sclerosis (MS) are accompanied by increased production of both TNFα and IFNγ^127,128^. TNFα induces apoptotic death in oligodendrocytes^129^ and also decreases the uptake of glutamate by astrocytes, which may further damage the oligodendrocytes^130^.

Multiple studies show that reactive oxygen species (ROS) appear to play an important role in the pathogenesis of neuro-inflammatory conditions^62,63,90^. Our results show that PFB, also a potent antioxidant, reduces TNFα, ROS and RANTES release from IL-1β-stimulated astrocytes. RANTES and its related receptors play a key role in the pathogenesis of Alzheimer’s disease^96^. Patients with AD have elevated levels of RANTES and its receptors (CCR5) in their brains^93,96^. AD lesions in the brain are associated with RANTES and its receptors. Multiple studies have suggested that RANTES indirectly mediates neuronal injury^128^. Some studies suggest that upregulation of RANTES may have a neuroprotective role^131–135^.

The clinical severity of Parkinson’s disease (PD) correlates with the serum level of RANTES^132^. The antibody neutralization of RANTES in a mouse model of Parkinson’s disease, MPTP-intoxicated mice, prevents the loss of dopaminergic neurons^136^. Patients with amyotrophic lateral sclerosis (ALS) have elevated levels of RANTES in their serum and cerebrospinal fluid^100^. The RANTES chemokine has been detected in brain lesions of patients with MS^134^. The CSF level of RANTES correlates well with the degree of neuroinflammation and the cortical synaptic excitability in patients with multiple sclerosis^134,137^.

In Alzheimer’s disease, the astrocytes express elevated levels of IP-10^138^. Injecting Aβ peptide into the mouse brain causes causes a marked increase in the secretion of IP-10 by the astrocytes^139^. Activated human astrocytes show enhanced production of IP-10, and both IP-10 and CXCR3 are upregulated in the brains of patients with AD^138^. The IP-10 levels correlate well with the clinical severity of disease in Parkinson’s patients^102^. Alpha-synuclein further increases the expression of IP-10 in the context of IL-1β-stimulated astroglial culture^140^. IP-10 has been demonstrated to be toxic to neurons, such as in the cell line LAN-2 and mixed human fetal neurons^141^.

IP-10 is also elevated in the CSF of MS patients^137^. Astrocytes in MS lesions express higher levels of IP-10^137,142^. Both serum and CSF levels of IP-10 are higher in MS patients with acute disease compared with those with stable disease^103,137,143^.

In summary, our data show that PFB significantly reduces the secretion of TNFα, RANTES and IP-10 by normal human astrocytes which have been activated by IL-1β. These 3 cytokines/chemokines belong to the early-prolonged response temporal group, which increase rapidly in the first 24 hours of exposure to PFB and remain elevated for at least 96 hours. There is extensive research literature which shows the importance of these 3 cytokines/chemokines in the pathogenesis and progression of neurodegenerative diseases including Alzheimer’s disease, Parkinson’s disease, Multiple sclerosis, Amyotrophic lateral sclerosis, HIV-induced dementia and several viral encephalitides.

Thus, TNFα, RANTES and IP-10 along with their receptors provide potential targets for drug intervention in combatting chronic neurodegenerative conditions due to the pivotal role these cytokines/chemokines play in the signaling networks, NF-κB versus Nrf2 transcriptional pathways and the known astrocyte-neuron system dynamics. Therefore, the effects of PFB on reducing these early-prolonged response cytokines/chemokines suggest that PFB may be effective in preventing or delaying the progression of the neuro-inflammation mediated by activated astrocytes. Based on this data, PFB may play a potentially beneficial role in Alzheimer’s, Parkinson’s, MS and ALS via its reduction of the pro-inflammatory cytokines TNFα, IP-10 and RANTES. The secretion of these three cytokines is transcriptionally regulated by NF-κB in astrocytes^53^. Our prior studies show that PFB downregulates the expression of the transcription factor NF-κB^80^, which may explain the reduction in expression of TNFα, IP-10 and RANTES by PFB in the IL-1β-stimulated astrocytes.

When comparing the phenolic versus the non-phenolic fractions of PFB, our data show that the non-phenolics fraction shows similar potency as the unfractionated PFB. The phenolics fraction shows no significant effect on TNFα, moderate effect on IP10 reduction, and the greatest effect on RANTES reduction, but still less than the unfractionated PFB. This suggests that other compounds play a greater role in this cytokine/chemokine reduction than do the phenolics fraction of PFB or that there may be some combinatorial/synergistic effect between the phenolics and non-phenolics in the PFB mixture.

Activated astrocytes and microglia, which produce ROS and NO, are seen in the substantia nigra of PD brains^144^. Usually mammalian cells will degrade physiologic levels of ROS/RNS through enzymatic reactions involving native cellular antioxidants/reducing agents such as glutathione. However, increased oxidative stress generates excessive levels of peroxyl radicals, hydroxyl radicals, superoxide anions, and peroxynitrites which cannot be easily degraded and require the consumption of direct antioxidants to scavenge them.

Prior studies of PFB measured the free radical scavenging activity using the 2,2-diphenyl-1-picrylhydrazyl (DPPH) reagent^71,73,75–77^. When activated by misfolded proteins, such as amyloid-β and α-synuclein, astrocytes generate ROS/RNS, which cause significant oxidative stress resulting in the significant neuronal injury and death underlying the pathology of the neuroinflammatory and neurodegenerative diseases^145–148^. The activated astrocytes produce excessive levels of ROS and NO in the CNS.

Our results show that PFB reduces the intracellular levels of ROS in activated astrocytes as measured by the DCDFA assay. This study shows that PFB has no effect on the basal level of ROS in resting astrocytes. IL-1β induces dose-dependent increases in the levels of ROS during astrocyte activation. PFB causes a dose-dependent reduction in the intracellular levels of ROS in activated astrocytes. There are 2 possible explanations for this reduction of ROS. PFB may directly act as a scavenger for ROS, as shown in prior studies. PFB may modulate the activity of superoxide dismutase (SOD), which facilitates the breakdown of free radical superoxide and hydrogen peroxide^147^, which plays an important role in maintaining the normal cellular redox status^148^.

The neuroinflammatory process seen in neurodegenerative diseases is characterized by the activation of astrocytes, which show increased expression of ICAM along with the secretion of pro-inflammatory cytokines^60,61^. The cellular adhesion molecules, upregulated in IL-1β-activated astrocytes, including ICAM, VCAM and NCAM, are potential biomarkers for the detection of the neuroinflammation seen in the neurodegenerative diseases^59^.

Studies show that reduction of the level of expressed ICAM on activated astrocytes correlates with reduced levels of aggregated beta-amyloid in Alzheimer’s disease^3^. The pro-inflammatory cytokine IL-1β causes increased levels of expression of ICAM in the CNS^149,150^. We show that PFB down-regulates the expression of ICAM on IL-1β-activated human astrocytes *in vitro* in a dose-dependent and time-dependent manner and this is accompanied by the reduced secretion of cytokines and chemokines. These cytokines and chemokines normally play an important role in the neuronal injury seen during neuroinflammation.

Our data also show that PFB reduces the secretion of sICAM and sVCAM. This is further evidence that PFB causes modulation and down-regulation in the activation of human astrocytes *in vitro*. The activation of astrocytes is a key process underlying the neuroinflammatory processes seen in the neurodegenerative diseases. Prior studies by our group showed that PFB also blocks the TNF-induced activation of NF-κB (Kang, unpublished data), and thus it is possible that PFB suppresses the expression of these adhesion molecules by inhibiting the activation of the pro-inflammatory transcription factor NF-κB in response to TNFα, and LPS. Similar concentrations of PFB block the expression of adhesion molecules and inhibit the expression of NF-κB thus suggesting a common pathway as the mechanism

Prior studies by our group showed that PFB inhibits the expression of ICAM-1, VCAM-1, and E-selectin on human umbilical vein endothelial cells which were stimulated with either TNFα or IL-1β, as well as inhibits the expression of NF-κB (Weinberg and Koledova, unpublished data). Furthermore RNA-seq experiments by our group show that PFB augments the expression of Nrf2 in astrocytes extracted from a human astrocytoma (Park, unpublished data). The reciprocal effects of PFB on NF-κB and Nrf2 make sense considering the homeostatic balance between the NF-κB pathway which usually activates pro-inflammatory genes and the Nrf2 pathway which usually activates anti-inflammatory genes^20,56,62^.

Some literature suggests that the pathways resulting in increased expression of ICAM-1 and E-selectin may be less responsive to antioxidants^146–148^. Other studies suggest that reactive oxygen intermediates activate NF-κB by a tyrosine kinase-dependent mechanism^149,151^. One possible mechanistic explanation for our results is that PFB may act to inhibit a specific protein tyrosine kinase that is upstream of IkBα kinase. This hypothesis will be the subject of future studies.

Studies also show that adhesion molecules such as ICAM are associated with the increased expression of RANTES comprising a direct correlation between the levels of RANTES levels and the levels of these adhesion molecules^150^. Consistent with these studies, our data show that PFB reduces both the secretion of RANTES and the expression of cell adhesion molecules in activated human astrocytes.

**Figure 8:**
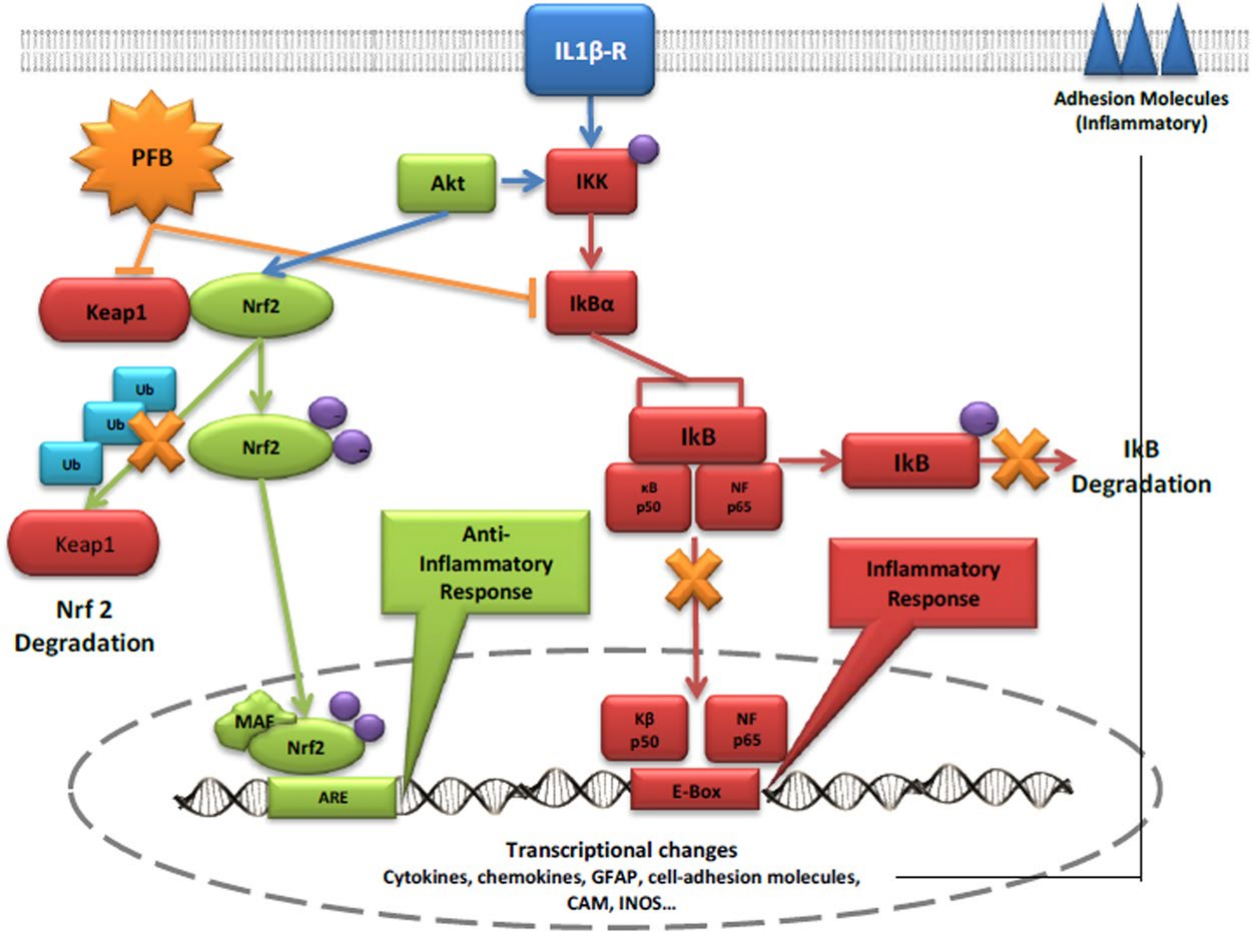
Pro-inflammatory and anti-inflammatory pathways potentially affected by PFB. The pathways indicated by the red color represent the pro-inflammatory pathways mediated by NF-κB. The pathways indicated by the green color represent the anti-inflammatory pathways mediated by Nrf2.

The cellular network of counterbalancing pro-inflammatory and anti-inflammatory pathways is depicted in Fig. 8. One mechanistic explanation of the actions of PFB on the dynamics of these pro-inflammatory and anti-inflammatory pathways is shown by the antagonistic responses of the NF-κB-mediated pro-inflammatory cascade to PFB, which are balanced against the Nrf2-mediated anti-inflammatory cascade mediated by PFB.

Our future research plans are focused on determining mechanistically where PFB and its components affect specific interactions in this network of anti-inflammatory and pro-inflammatory pathways seen in Fig. 8.

## Conclusions

The data show that the cytokine/chemokine profiles of basal unstimulated astrocytes are different and distinct from the cytokine/chemokine profile of the IL-1β activated astrocytes. We called these 2 profiles the normal and the inflammatory cytokine/chemokine profiles. Furthermore, the cytokine/chemokine profiles fall into 3 temporal groups according to the timing from IL-1β exposure to maximal response: the early response, early prolonged response and the delayed response.

This study shows the multiple mechanisms by which PFB acts: (1) decreasing the expression of pro-inflammatory cytokines/chemokines TNFα, RANTES and IP-10, which belong to the early prolonged response group and play a key role in chronic inflammation and the progression of neurodegenerative diseases; (2) reducing the oxidative stress in the inflammatory response by reducing the intracellular levels of ROS; (3) reducing the expression of inflammation-associated cell-surface adhesion molecules ICAM and VCAM which are important in recruiting the adaptive immune response; and (4) reducing the cytokine/chemokine profile differentially by the phenolic and non-phenolic fractions of PFB.

The observed anti-inflammatory and neuroprotective effects of PFB on astrocyte reactivity suggest therapeutic potential of PFB for neuroinflammatory conditions. PFB attenuates the production of pro-inflammatory cytokines and chemokines as well as reduces the expression of ROS and cellular adhesion molecules (CAM). Our study suggests that PFB causes a significant reduction in astrocyte activation with concomitant reduction of cytokine/chemokine production, ROS production and CAM expression. In summary, these PFB-induced changes suggest the potential that PFB may have in protecting against the neuro-inflammatory processes observed in the neurodegenerative conditions. PFB potentially may prevent astrocyte-mediated neuroinflammatory injury to the brain. These results show that PFB has important immunomodulatory effects on IL-1β-activated human astrocytes *in vitro*, which suggests that PFB may have a beneficial effect in the prevention and/or treatment of the neuroinflammatory state associated with such neurodegenerative diseases as Alzheimer’s Disease, Parkinson’s Disease, and Huntington’s Disease, among others^152–158^.

## Electronic supplementary material

Supplementary Figures and Tables

## Data Availability

The datasets and supporting information of this article are included within the article and its additional supporting files.
