# Supplementary material for: Palm Fruit Bioactives modulate human astrocyte activity in vitro altering the cytokine secretome reducing levels of TNFα, RANTES and IP-10
Source: Sci Rep. 2018 Nov 6;8:16423. doi: 10.1038/s41598-018-34763-3 (PMC6219577; doi:10.1038/s41598-018-34763-3)
Supplement: Supplementary file 1 — Supplementary Figures and Tables [file 41598_2018_34763_MOESM1_ESM.pdf]

Additional Figures and Tables

Palm Fruit Bioactives modulate human astrocyte activity *in vitro* altering the cytokine secretome reducing levels of TNFα, RANTES and IP-10

Robert P. Weinberg†[1,2*], Vera V. Koledova†[1,2], Kirsten Schneider[1,2], TG Sambandan[1,2], Adlai Grayson[1,2], Gal Zeidman[1,2], Anastasia Artamonova[1,2], Ravigadevi Sambanthamurthi[3], Syed Fairus[3], Anthony J. Sinskey[1,2], ChoKyun Rha[2*]

† Authors RPW and VVK contributed equally to this paper

Addresses: [1]Department of Biology, Massachusetts Institute of Technology, Cambridge, MA 02139, USA; [2]Biomaterials Science and Engineering Laboratory, Massachusetts Institute of Technology, Cambridge, MA 02139, USA; [3]Advanced Biotechnology and Breeding Centre, Malaysian Palm Oil Board, 6, Persiaran Institusi, Bandar Baru Bangi, 43000 Kajang, Selangor, Malaysia

Emails: Robert P. Weinberg* - rweinber@mit.edu; Vera V. Koledova - koledova@mit.edu; Kirsten Schneider – kirstens@mit.edu; TG Sambandan – gnana@mit.edu; Adlai Grayson - adlai.grayson@bison.howard.edu; Gal Zeidman - gzeidman@mit.edu; Anastasia Artamonova – artana@mit.edu; Ravigadevi Sambanthamurthi – ravigadevi@gmail.com; Syed Fairus - syfairus@mpob.gov.my; Anthony Sinskey – asinskey@mit.edu; ChoKyun Rha* – ckrha@mit.edu

*Corresponding authors: Robert Weinberg – rweinber@mit.edu
ChoKyun Rha – ckrha@mit.edu

Proof and Reprint Requests should be addressed to:

Robert P. Weinberg
Department of Biology
Biomaterials Science and Engineering Laboratory
Massachusetts Institute of Technology
77 Massachusetts Avenue, Room 56-265A
Cambridge, Massachusetts 02139 U.S.A.
(Telephone) +1-617-253-3493
(Facsimile) +1-617-253-6358
(Email) rweinber@mit.edu

Captions to Supplementary Figures and Tables

Additional Figure 1.

File format: .docx

Title: Additional Figure 1. GFAP expression by normal human astrocytes (NHA) shown by immunohistochemistry.

Description: GFAP-positive astrocytes have intensely brown cytoplasmic stain, nuclei are counterstained with haematoxylin (blue). Positive GFAP staining was ~80 percent. Primary GFAP-antibodies obtained from Santa-Cruz Biotechnology.

Additional Table 1.

File format: .docx.

Title: Additional Table 1. Absolute values for cytokine/chemokine secretion by human astrocytes with stimulation by IL-1β at 24 and 96 hours.

Description: For each cytokine, values are given as average concentration +/- standard deviation for the IL-1β-stimulated condition. ND indicates not detected, outside the limits of quantitation. n/A for standard deviation indicates only one measurement was within the range of quantitation. Experiment performed in triplicate.

Additional Table 2.

File format: .docx

Title: Additional Table 2. Absolute values for cytokine/chemokine secretion by unstimulated human astrocytes at 24 hours exposed to PFB.

Description: For each cytokine, values are given as average concentration +/- standard deviation. ND indicates not detected. n/A for standard deviation indicates only one measurement was within the range of quantitation. Experiment performed in triplicate.

Additional Table 3.

File format: .docx

Title: Additional Table 3. Absolute values for cytokine/chemokine secretion by unstimulated human astrocytes at 96 hours exposed to PFB.

Description: For each cytokine, values are given as average concentration +/- standard deviation. ND indicates not detected. n/A for standard deviation indicates only one measurement was within the range of quantitation. Experiment performed in triplicate.

Additional Table 4.

File format: .docx

Title: Additional Table 4. Palm Fruit Bioactives significantly decreased secretion of cytokines RANTES, TNFα, and IP-10, inflammatory markers induced by IL-1β in human astrocytes in the present study.

Description: IL-1β stimulation and Palm Fruit Bioactives treatment were performed simultaneously, with media harvest occurring at 24 and 96 hours. The experiment was performed three times on subsequent passages of NHA in duplicate for each passage (experiment N1, N2, and N3). Means of individual experiments are listed with the measurement standard deviation; despite large inter-experimental variation of means, PFB decreased expression of these inflammatory markers in a dose-dependent manner in all experiments. ND indicates below the level of quantitation, n/A for standard deviation indicates only one measurement was within the range of quantitation.

Additional Figure 2.

File format: .docx

Title: Additional Figure 1. Different fraction of PFB as shown by chromatograms of HPLC.

Description: A) Phenolics fraction of PFB contains five marker compounds, protacatechuic acid, 4-hydroxybenzoic acid, and three caffeoylshikimic acid isomers, among others; B) non-phenolics fraction of PFB does not possess phenolic acid marker compounds and does possess soluble fibers, intercalated shikimic acid, and other relatively polar compounds. Fractionation performed by ethyl acetate and water liquid-liquid extraction.

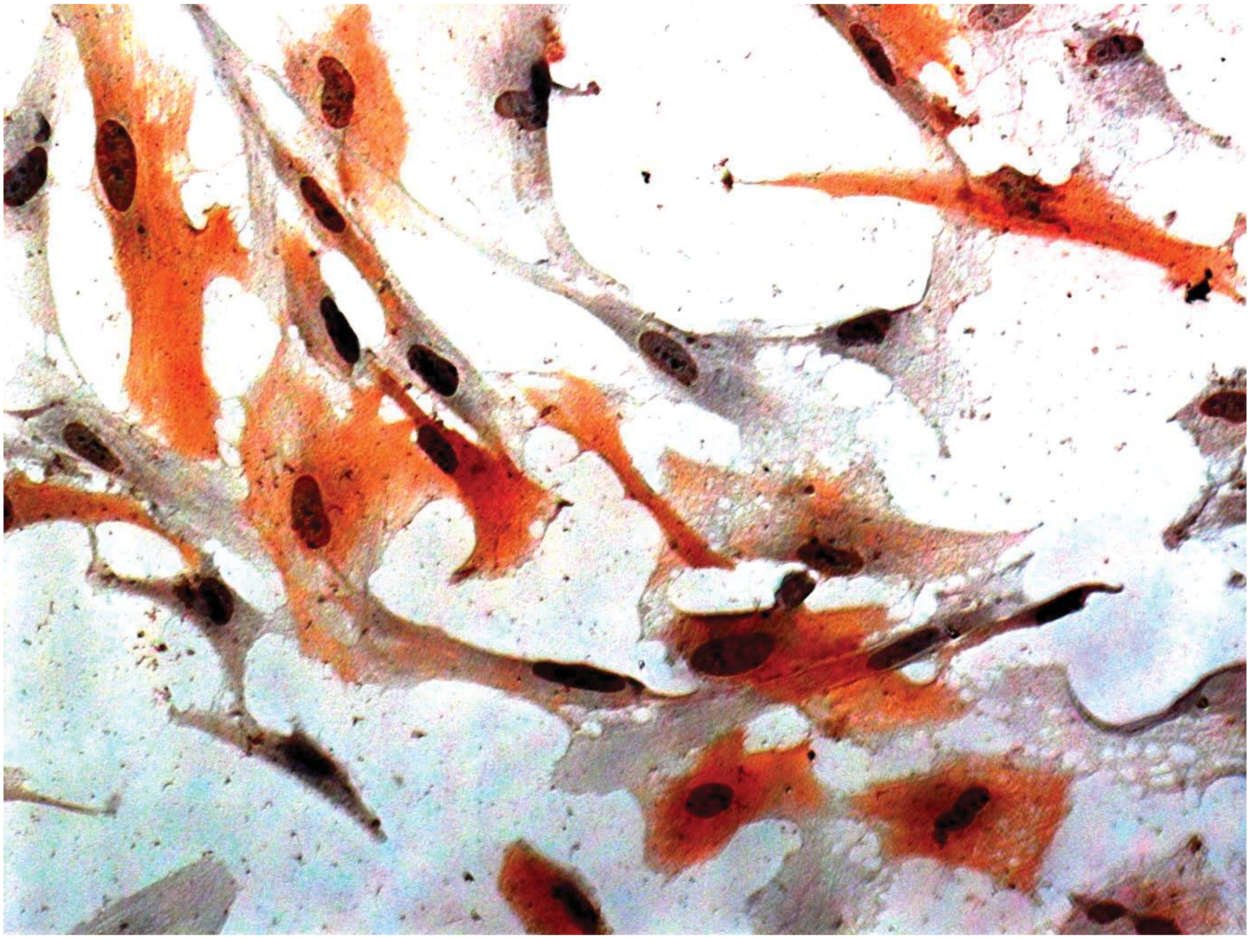

Additional Figure 1.

File format: .docx

Title: Additional Figure 1. Normal Human Astrocytes (NHA), immunohistochemistry staining for GFAP (brown color), haematoxylin counterstain for nuclei (blue).

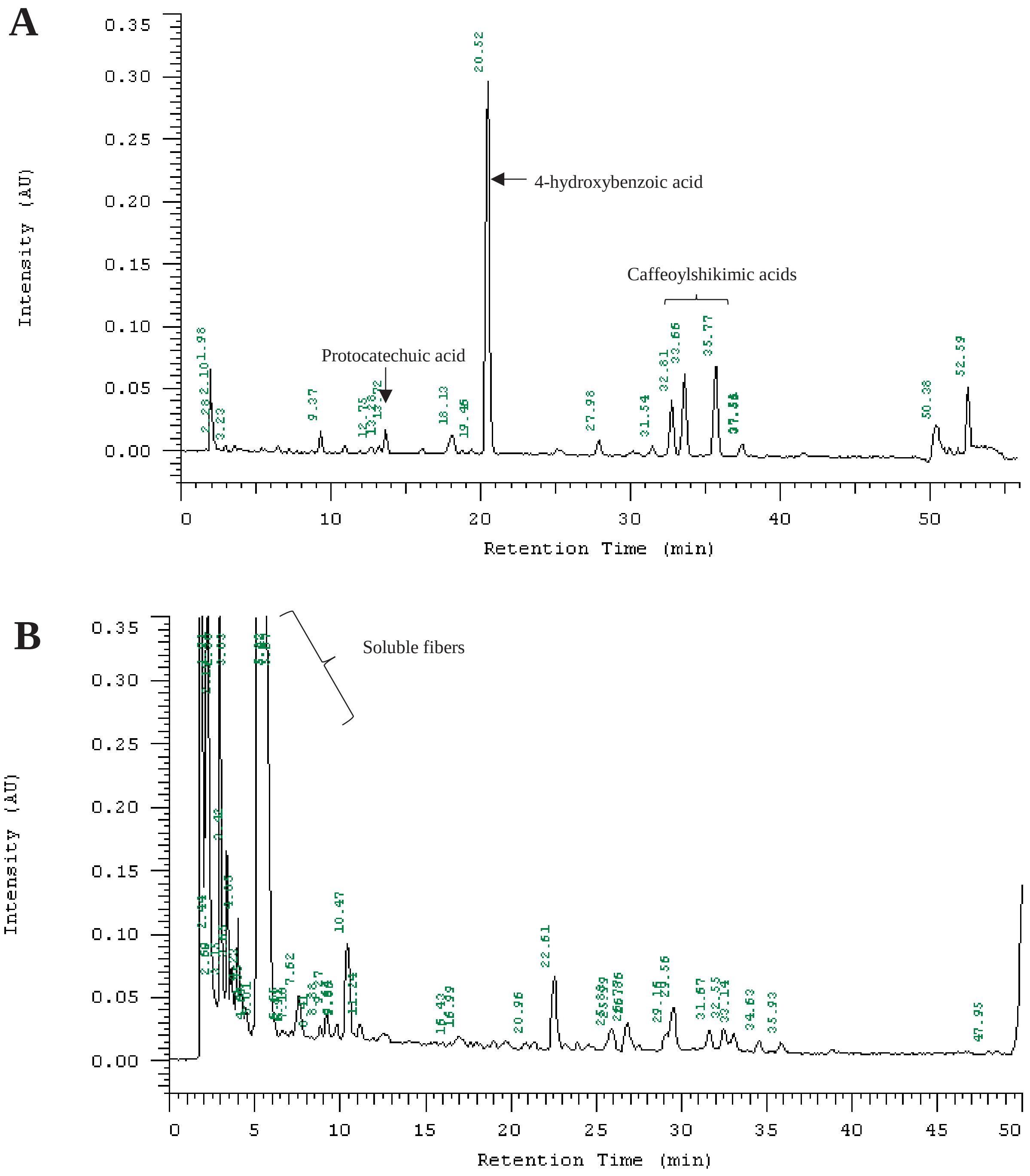

Additional Figure 2

Additional Table 1. Absolute values for cytokine/chemokine secretion by human astrocytes with stimulation by IL-1β at 24 and 96 hours.

Protein	Gene location that encodes protein	24 h protein concentration Avg ± 1 STD (pg/mL)	96 h protein concentration Avg ± 1 STD (pg/mL)
Eotaxin	17q12	33.1 ± 6.7	48.1 ± 19.0
FGF-2	4q28.1	57.5 ± 20.3	76.7 ± 33.4
Flt3L	19q13.33	ND	ND
Fractalkine	16q21	136.6 ± 29.4	205.8 ± 31.7
G-CSF	17q21.1	5906.5 ± 3503.5	10993.5 ± 6473.6
GM-CSF	5q31.1	2587.2 ± 1309.7	6902.3 ± 2359.7
GRO	4q13.3	15553.0 ± n/A	14730.0 ± n/A
IFNα2	9p21.3	42.1 ± 18.2	55.1 ± 11.4
IFNγ	12q15	10.6 ± 1.4	7.6 ± 5.6
IL-10	1q32.1	ND	ND
IL-12(p40)	5q33.3	10.3 ± 2.7	11.9 ± 6.9
IL-12(p70)	3Q25.33 & 5q33.3	10.4 ± 5.2	9.0 ± 4.2
IL-13	5q31.1	5.4 ± 0.8	7.1 ± 3.0
IL-15	4q31.21	3.9 ± 0.7	11.5 ± 5.2
IL-17A	6p12.2	4.5 ± 2.1	5.1 ± 1.9
IL-1α	2q14.1	7.3 ± 1.6	29.7 ± 10.4
IL-1RA	2q14.1	215.2 ± 341.7	14.5 ± 14.5
IL-2	4q27	ND	ND
IL-3	5q31.1	ND	ND
IL-4	5q31.1	15.6 ± 2.0	21.9 ± 8.1
IL-5	5q31.1	ND	ND
IL-6	7p15.3	13416.3 ± 4595.6	15942.0 ± 2545.6
IL-7	8q21.13	24.8 ± 17.9	32.6 ± 7.7
IL-8	4q13.3	14453.0 ± 145.7	11357.5 ± 7139.7
IL-9	5q31.1	ND	ND
IP-10	4q21.1	561.9 ± 313.7	2749.9 ± 1255.7
MCP-1	17q12	ND	ND
MCP-3	17q12	62.5 ± 71.6	530.3 ± 89.7
MDC	16q21	6.4 ± 1.1	6.3 ± 2.4
MIP-1α	17q12	134.9 ± 198.8	500.4 ± 737.5
MIP-1β	17q12	88.3 ± 75.7	94.2 ± 29.7
PDGF-AA	7p22.3	131.0 ± 125.9	2668.7 ± 2456.1
PDGF-AB/BB	7p22.3 & 22q13.1 or 22q13.1 & 22q13.1	24.4 ± 18.8	223.5 ± 138.2
RANTES	17q12	1565.6 ± 1403.3	5063.2 ± 2129.5
sCD40L	Xq26.3	9.1 ± 4.5	12.3 ± 3.7
TGFα	2p13.3	53.7 ± 59.2	122.6 ± 22.7
TNFα	6p21.33	706.2 ± 1114.5	183.9 ± 20.3
TNFβ	6p21.33	4.5 ± 1.8	4.7 ± 2.2
VEGF	6p21.1	84.2 ± 12.7	976.3 ± n/A

ND indicates not detected, outside the limits of quantitation. n/A for standard deviation indicates only one measurement was within the range of quantitation. Experiment performed in triplicate.

Additional Table 2. Absolute values for cytokine/chemokine secretion by unstimulated human astrocytes at 24 hours exposed to PFB.

Cytokine/chemokine profile for unstimulated NHA at 24 h exposed to PFB					
Protein	Gene location that encodes protein	Untreated Protein concentration Avg ± SD (pg/mL)	PFB 10 μL/mL exposure Protein concentration Avg ± SD (pg/mL)	PFB 20 μL/mL exposure Protein concentration Avg ± SD (pg/mL)	PFB 40 μL/mL exposure Protein concentration Avg ± SD (pg/mL)
Eotaxin	17q12	4.5 ± 0.0	8.3 ± 5.3	4.6 ± 0.1	6.6 ± 0.8
FGF-2	4q28.1	< 40.5	25.1 ± n/A	21.7 ± n/A	23.4 ±
Flt3L	19q13.33	ND	ND	ND	ND
Fractalkine	16q21	54.1 ± 17.2	66.1 ± 14.9	34.7 ± 22.7	52.4 ± 1.7
G-CSF	17q21.1	10.4 ± 7.7	11.8 ± 7.1	7.6 ± 4.8	10.6 ± 0.8
GM-CSF	5q31.1	4.5 ± 1.8	4.9 ± 1.5	3.5 ± 0.9	3.6 ± 0.5
GRO	4q13.3	78.8 ± 40.9	88.7 ± 51.7	83.2 ± 49.0	78.6 ± 58.4
IFNα2	9p21.3	9.1 ± 2.7	13.0 ± 5.4	5.8 ± n/A	7.0 ± 1.3
IFNγ	12q15	4.4 ± 2.2	5.4 ± 2.8	1.4 ± n/A	3.7 ± n/A
IL-10	1q32.1	ND	ND	ND	ND
IL-12(p40)	5q33.3	6.2 ± 3.6	9.9 ± 4.7	3.3 ± 0.1	6.4 ± 0.8
IL-12(p70)	3Q25.33 & 5q33.3	< 3.2	3.6 ± 0.0	2.9 ± n/A	1.9 ± n/A
IL-13	5q31.1	2.3 ± 1.6	1.5 ± n/A	1.2 ± n/A	1.2 ± n/A
IL-15	4q31.21	2.2 ± 1.1	2.2 ± 0.6	1.3 ± 1.7	2.4 ± 1.4
IL-17A	6p12.2	ND	2.5 ± 1.2	0.3 ± n/A	0.9 ± n/A
IL-1α	2q14.1	< 2.7	3.0 ± 1.0	1.3 ± n/A	2.2 ± n/A
IL-1RA	2q14.1	ND	324.8 ± 454.8	ND	353.1 ± 494.1
IL-2	4q27	ND	ND	ND	ND
IL-3	5q31.1	ND	ND	ND	ND
IL-4	5q31.1	ND	2.6 ± n/A	3.9 ± 2.1	4.6 ± n/A
IL-5	5q31.1	ND	ND	ND	ND
IL-6	7p15.3	579.2 ± 312.4	848.8 ± 261.3	979.6 ± 182.5	1001.2 ± 307.3
IL-7	8q21.13	<3.2	6.2 ± 4.0	ND	ND
IL-8	4q13.3	147.6 ± 72.6	192.9 ± 149.4	150.8 ± 105.4	144.6 ± 83.4
IL-9	5q31.1	ND	ND	ND	ND
IP-10	4q21.1	28.0 ± 17.0	29.0 ± 34.5	2.9 ± n/A	22.4 ± 27.2
MCP-1	17q12	ND	ND	ND	ND
MCP-3	17q12	20.1 ± 2.2	14.4 ± n/A	13.6 ± 5.1	13.8 ± 0.7
MDC	16q21	4.8 ± 2.5	4.9 ± 1.0	4.0 ± 1.3	3.8 ± 0.6
MIP-1α	17q12	27.1 ± 0.5	15.4 ± 7.5	19.7 ± n/A	ND
MIP-1β	17q12	3.9 ± n/A	3.4 ± 0.9	1.9 ± n/A	2.8 ± 0.1
PDGF-AA	7p22.3	155.5 ± 7.5	133.6 ± 67.6	114.2 ± 63.2	104.5 ± 36.1
PDGF-AB/BB	7p22.3 & 22q13.1 or 22q13.1 & 22q13.1	< 22.0	20.3 ± 22.8	12.5 ± 12.5	8.7 ± 8.4
RANTES	17q12	4.0 ± n/A	4.7 ± 1.7	6.3 ± n/A	5.5 ± n/A
sCD40L	Xq26.3	8.2 ± 3.3	9.7 ± 2.9	7.3 ± 0.0	9.6 ± 2.2
TGFα	2p13.3	40.4 ± 30.3	38.9 ± 19.6	35.1 ± 21.0	35.9 ± 15.8
TNFα	6p21.33	3.2 ± 0.0	2.2 ± 1.2	1.5 ± 1.6	1.9 ± 1.6
TNFβ	6p21.33	ND	2.0 ± 0.9	1.7 ± 1.4	1.3 ± n/A
VEGF	6p21.1	ND	11.1 ± n/A	ND	13.7 ± n/A

ND indicates not detected. n/A for standard deviation indicates only one measurement was within the range of quantitation. Experiment performed in triplicate.

Additional Table 3. Absolute values for cytokine/chemokine secretion by unstimulated human astrocytes at 96 hours exposed to PFB.

Cytokine/chemokine profile for unstimulated NHA at 96 h exposed to PFB					
Protein	Gene location that encodes protein	Untreated Protein concentration Avg ± SD (pg/mL)	PFB 10 μL/mL exposure Protein concentration Avg ± SD (pg/mL)	PFB 20 μL/mL exposure Protein concentration Avg ± SD (pg/mL)	PFB 40 μL/mL exposure Protein concentration Avg ± SD (pg/mL)
Eotaxin	17q12	31.1 ± 28.8	32.7 ± 13.4	15.2 ± 3.7	6.2 ± 1.7
FGF-2	4q28.1	68.6 ± 26.5	40.6 ± 3.9	39.2 ± 17.4	34.1 ± n/A
Flt3L	19q13.33	ND	ND	ND	ND
Fractalkine	16q21	101.1 ± 49.1	94.2 ± 25.0	64.3 ± 22.1	52.0 ± 23.3
G-CSF	17q21.1	463.1 ± 561.2	37.5 ± 11.2	21.4 ± 8.2	43.5 ± 11.9
GM-CSF	5q31.1	253.1 ± 376.1	16.2 ± 13.1	12.6 ± 3.3	9.6 ± 3.6
GRO	4q13.3	699.6 ± 486.3	593.7 ± 297.1	428.3 ± 57.9	279.9 ± 138.2
IFNα2	9p21.3	23.3 ± 9.4	24.5 ± 2.7	15.7 ± 7.1	12.1 ± 1.5
IFNγ	12q15	5.7 ± 2.0	4.8 ± 0.6	5.5 ± 3.7	4.3 ± 1.3
IL-10	1q32.1	ND	ND	ND	ND
IL-12(p40)	5q33.3	8.8 ± 3.1	7.6 ± 1.1	6.6 ± 3.8	5.5 ± 1.9
IL-12(p70)	3Q25.33 & 5q33.3	6.4 ± 0.3	7.0 ± 3.0	4.9 ± 2.4	3.7 ± 0.3
IL-13	5q31.1	2.3 ± n/A	1.8 ± n/A	2.5 ± 0.8	1.5 ± n/A
IL-15	4q31.21	5.8 ± 4.9	5.4 ± 1.9	3.9 ± 1.9	3.8 ± 1.7
IL-17A	6p12.2	3.3 ± 0.3	2.5 ± 1.7	2.5 ± 1.5	2.8 ± 0.8
IL-1α	2q14.1	8.2 ± 1.3	2.7 ± 0.6	4.4 ± 2.7	5.4 ± 1.8
IL-1RA	2q14.1	18.2 ± n/A	2923.2 ± 4127.8	2066.2 ± 2917.3	878.3 ± 1236.9
IL-2	4q27	ND	ND	ND	ND
IL-3	5q31.1	ND	ND	ND	ND
IL-4	5q31.1	18.9 ± n/A	2.9 ± 1.1	ND	ND
IL-5	5q31.1	ND	ND	ND	ND
IL-6	7p15.3	3980.1 ± 3442.9	5384.6 ± 3356.6	4961.3 ± 2604.1	2000.3 ± 273.1
IL-7	8q21.13	29.5 ± 3.5	12.1 ± 3.6	6.6 ± 2.5	3.4 ± 1.5
IL-8	4q13.3	1581.5 ± 1289.7	2233.6 ± 1407.0	1279.7 ± 543.5	717.6 ± 92.2
IL-9	5q31.1	ND	ND	ND	ND
IP-10	4q21.1	34.0 ± 20.7	15.9 ± 15.8	21.0 ± 21.3	30.5 ± 38.6
MCP-1	17q12	ND	8262.0 ± n/A	ND	ND
MCP-3	17q12	82.6 ± 79.7	50.3 ± 34.5	28.0 ± 5.8	14.2 ± 4.3
MDC	16q21	5.4 ± 1.6	8.0 ± 3.9	5.7 ± 2.2	5.6 ± 2.5
MIP-1α	17q12	24.4 ± 5.0	21.5 ± 14.3	16.6 ± 8.4	19.2 ± 13.3
MIP-1β	17q12	18.2 ± 8.8	11.7 ± 1.6	6.1 ± 3.1	3.5 ± 1.4
PDGF-AA	7p22.3	2183.2 ± 2002.0	1289.2 ± 824.5	994.4 ± 567.5	401.1 ± 135.2
PDGF-AB/BB	7p22.3 & 22q13.1 or 22q13.1 & 22q13.1	157.7 ± 110.0	45.9 ± 21.0	19.9 ± 20.8	13.3 ± 10.1
RANTES	17q12	22.2 ± 12.6	4.1 ± 1.4	5.6 ± 2.3	6.2 ± 5.4
sCD40L	Xq26.3	5.9 ± 2.6	7.5 ± 3.2	8.6 ± 0.1	8.8 ± 1.9
TGFα	2p13.3	63.6 ± 43.1	82.7 ± 37.5	128.1 ± 36.4	138.9 ± 38.3
TNFα	6p21.33	18.0 ± 17.5	1.9 ± 2.0	3.5 ± 1.3	2.1 ± 2.1
TNFβ	6p21.33	3.2 ± 0.8	2.6 ± n/A	2.4 ± n/A	1.1 ± n/A
VEGF	6p21.1	ND	124.5 ± n/A	12.0 ± n/A	161.3 ± n/A

ND indicates not detected. n/A for standard deviation indicates only one measurement was within the range of quantitation. Experiment performed in triplicate.

Additional Table 4. Palm Fruit Bioactives significantly decreased secretion of cytokines RANTES, TNFα, and IP-10, inflammatory markers induced by IL-1β in human astrocytes in the present study.

24 hour experiment – IL-1β stimulated					
Analyte	Experiment	PFB 0 μL/mL Avg ± SD (pg/mL)	PFB 10 μL/mL Avg ± SD (pg/mL)	PFB 20 μL/mL Avg ± SD (pg/mL)	PFB 40 μL/mL Avg ± SD (pg/mL)
RANTES	N1	407.3 ± 12.4	305 ± 26.1	114.8 ± 7.1	38.1 ± 6.6
	N2	2943.5 ± 358.5	1927.5 ± 277.9	1399.5 ± 88.4	1064.4 ± 99.6
	N3	580.0 ± 24.7	673.2 ± 70.7	214.4 ± 8.7	12.0 ± n/A
TNFα	N1	91.2 ± 3.5	62.1 ± 3.0	46.6 ± 2.1	26.0 ± 2.7
	N2	222.0 ± 16.5	72.1 ± 14.1	50.6 ± 2.6	34.8 ± 1.7
	N3	136 ± 0.5	140.1 ± 16.9	104.3 ± 12	20.3 ± 3.4
IP-10	N1	885.2 ± 46.6	303.1 ± 31.2	96.5 ± 3.1	22.6 ± 5.1
	N2	757.1 ± 62.0	250.8 ± 20.1	116.7 ± 20.5	ND
	N3	396.4 ± 3.6	236.4 ± 15.8	114.5 ± 14.8	ND
96 hour experiment – IL-1β stimulated					
Analyte	Experiment	PFB 0 μL/mL Avg ± SD (pg/mL)	PFB 10 μL/mL Avg ± SD (pg/mL)	PFB 20 μL/mL Avg ± SD (pg/mL)	PFB 40 μL/mL Avg ± SD (pg/mL)
RANTES	N1	3900.5 ± 273.7	903.3 ± 37.5	204.1 ± 0.5	5 ± n/A
	N2	8465 ± 633.6	5854.5 ± 229.8	3629.5 ± 285.0	1326 ± 66.5
	N3	2844.5 ± 468.8	801.6 ± 157.2	458.5 ± 74.3	ND
TNFα	N1	200.6 ± 11.2	95.0 ± 0.7	44.3 ± 1.7	2.4 ± 0.4
	N2	190.0 ± 4.1	76.7 ± 0.5	51.7 ± 5.8	27.0 ± 0.3
	N3	161.4 ± 24.3	70.9 ± 12.1	90.7 ± 7.1	4.8 ± 2.4
IP-10	N1	3459 ± 355.0	1064.5 ± 6.4	323.9 ± 17.6	11.8 ± 1.8
	N2	961.0 ± 84.8	385.8 ± 0.8	146.3 ± 8.2	ND
	N3	2826 ± 538.8	855.8 ± 91.0	298.1 ± 56.3	50.7 ± 3.4

IL-1β stimulation and Palm Fruit Bioactives treatment were performed simultaneously, with media harvest occurring at 24 and 96 hours. The experiment was performed three times on subsequent passages of NHA in duplicate for each passage (experiment N1, N2, and N3). Means of individual experiments are listed with the measurement standard deviation; despite large inter-experimental variation of means, PFB decreased expression of these inflammatory markers in a dose-dependent manner in all experiments. ND indicates below the level of quantitation, n/A for standard deviation indicates only one measurement was within the range of quantitation.
